# An Impact Analysis of Artificial Light at Night (ALAN) on Bats. A Case Study of the Historic Monument and Natura 2000 Wisłoujście Fortress in Gdansk, Poland

**DOI:** 10.3390/ijerph182111327

**Published:** 2021-10-28

**Authors:** Karolina M. Zielinska-Dabkowska, Katarzyna Szlachetko, Katarzyna Bobkowska

**Affiliations:** 1 GUT LightLab, Faculty of Architecture, Gdansk University of Technology, 80-233 Gdansk, Poland; 2 Faculty of Law and Administration, University of Gdansk, 80-980 Gdansk, Poland; katarzyna.szlachetko@prawo.ug.edu.pl; 3 Faculty of Civil and Environmental Engineering, Gdansk University of Technology, 80-233 Gdansk, Poland; katarzyna.bobkowska@pg.edu.pl

**Keywords:** external illumination, sustainable urban lighting, artificial light at night, ALAN, anthropogenic light pollution, ecological light pollution, nighttime conservation, environmental protection, environmental pollution, bats, satellite remote sensing, light pollution regulations, Natura 2000, *Myotis dasycneme*, VIIRS, nighttime light observations, NTL, Black Marble, light pollution map, emissions control, COVID-19

## Abstract

The artificial light at night (ALAN) present in many cities and towns has a negative impact on numerous organisms that live alongside humans, including bats. Therefore, we investigated if the artificial illumination of the historic Wisłoujście Fortress in Gdańsk, Poland (part of the Natura 2000 network), during nighttime events, which included an outdoor electronic dance music (EDM) festival, might be responsible for increased light pollution and the decline in recent years of the pond bat (*Myotis dasycneme)*. An assessment of light pollution levels was made using the methods of geographical information system (GIS) and free-of-charge satellite remote sensing (SRS) technology. Moreover, this paper reviewed the most important approaches for environmental protection of bats in the context of ecological light pollution, including International, European, and Polish regulatory frameworks. The analysis of this interdisciplinary study confirmed the complexity of the problem and highlighted, too, the need for better control of artificial illumination in such sensitive areas. It also revealed that SRS was not the best light pollution assessment method for this particular case study due to several reasons listed in this paper. As a result, the authors’ proposal for improvements also involved practical recommendations for devising suitable strategies for lighting research and practice in the Natura 2000 Wisłoujście Fortress site located adjacent to urban areas to reduce the potential negative impact of ALAN on bats and their natural habitats.

## 1. Introduction 

Ecological light pollution (ELP) and the adverse impact of artificial light at night (ALAN) on nature have both been studied by environmental scientists over the years [1,2]. Many living organisms on planet Earth are nocturnal species that have a preference for nighttime activity which allows them to avoid predators and heat and have safer feeding and/or facilitates reproduction [3]. Despite growing concerns, it is commonplace for modern society to brightly illuminate nightscapes. Consequently, the crucial role of natural darkness and the importance of nocturnal placemaking have been undervalued, not only as critical components of the environment but also as requisites for ecosystems and biological processes for all living organisms.

**Figure A1:**
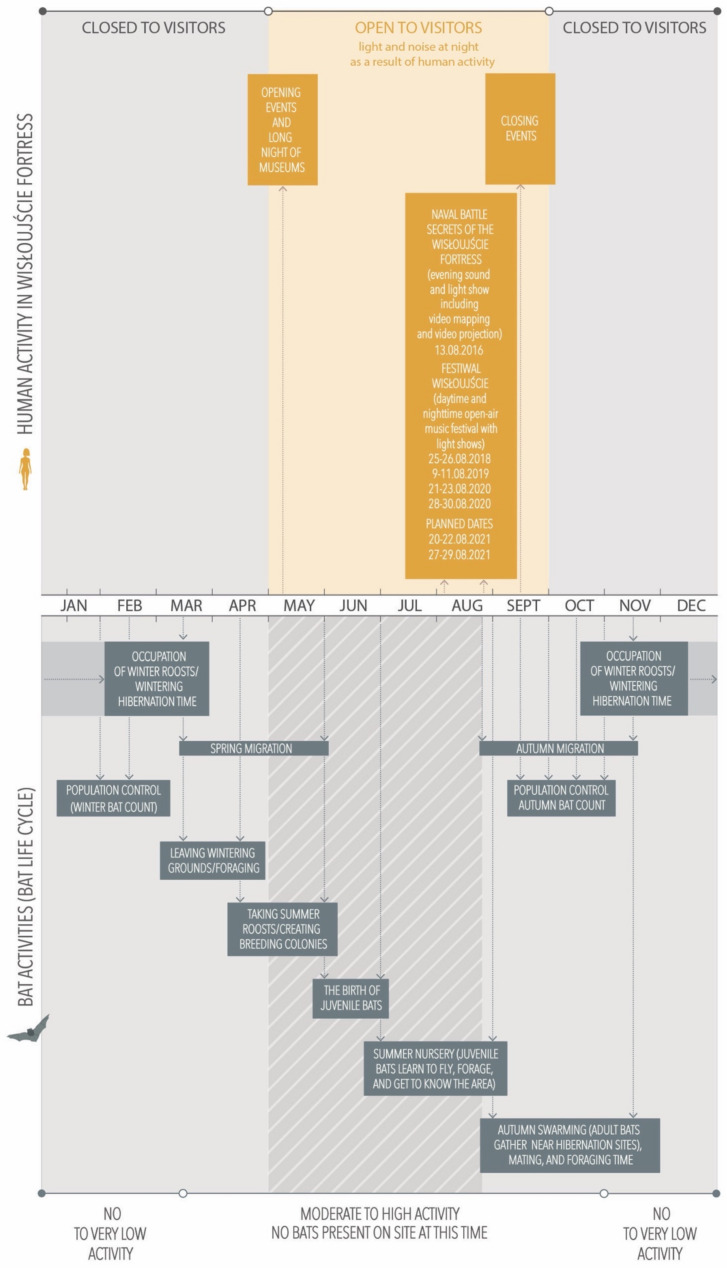
An overview of annual human and bat activities in the Natura 2000 Wisłoujście Fortress, Gdańsk, Poland. Source: authors’ elaboration based on [151].

To date, researchers have investigated ALAN and its possible impact on the environment from various perspectives including: measurement methods [4,5,6], regulatory frameworks [7,8], anthropogenic disruptions and ecological consequences [9,10,11,12,13,14], changes in ecosystems [15,16,17,18], hormonal changes in various species [19], public health [20,21], sustainability [22], celestial visibility [23,24], protected areas [25], environmental philosophy [26], and citizen actions against light pollution [27]. However, there is still very little research about the impact of ALAN from nighttime events, including outdoor music festivals, on environmentally sensitive areas [28]. Artificial lighting at night is often applied without legal constraints, even in protected sites such as those that have been given Natura 2000 status. This protection provides a sheltered habitat for Europe’s most valuable and threatened species. (For more information on Natura 2000, see Section 2.1 and Section 5.5) Most frequently, the habitats of endangered species, such as bats, are found near urban areas, and the light that emanates from built environments repeatedly disturbs their biological responses. Nevertheless, even when there are protection initiatives, festival and event lighting and the illumination of historic monuments have not been considered in detail. Thus, more studies are required to provide a clearer picture of the current situation. For instance, the daytime and nighttime annual electronic music Wisłoujście Festival held on the grounds of the Natura 2000 Wisłoujście Fortress site [29] uses excessive artificial lighting (these illuminance levels and the use of coloured light do not comply with EUROBATS guidelines). This event has occurred at the end of August since 2018 [30], when the autumn swarming of bats takes place on-site [31]. (For more information about the bat presence on-site, see Appendix A, Figure A1.) There has been an observed decrease in the number of endangered bat species *Myotis dasycneme* in Europe [32,33] and since 2016 in this particular Natura 2000 site, which raises the question of whether these bats receive appropriate protection when such events are permitted.

Our main aim was to ascertain the on-site temporal trend in light pollution over the decade, including the period of time during the COVID-19 lockdown of 2020, to determine whether light pollution might explain the decline of the endangered pond bat, and to evaluate the most suitable scientific method for such analysis for non-GIS and -SRS experts and the sufficiency of legal conditions for the protection of the Natura 2000 Wisłoujście Fortress in Poland.

With sensitive landscape areas such as Natura 2000 sites located in or adjacent to anthropogenic environments, this work is not only important and required, it will also help to protect the future of such nightscapes and improve the existing situation of bat species via guidance that adds new understanding to the existing research field. 

This paper is arranged into eight sections. Section 1 demonstrates how the research fits within a larger field of study and highlights why it is important in the context of natural environments and biodiversity. Section 2 provides a background study on *Myotis dasycneme* bats and the use of remote sensing as a possible tool for analysis. Section 3 defines the research questions. Section 4 describes the methods and protocols used in the study. Section 5 provides a precise description and summary of the achieved results. Section 6 discusses the findings and their consequences and explains how they can be decoded via the perspective of the research questions. Section 7 provides the study’s limitations. Lastly, Section 8 puts forward practical recommendations and suggests new necessary areas for future research on the topic. Appendix A, Appendix B, Appendix C and Appendix D offer extended information on the issues discussed to make this paper even more comprehensive.

## 2. Research Background

Extensive interdisciplinary background research was undertaken involving examining the study area and events on-site, investigating the life of bats including the pond bat (*Myotis dasycneme*), a thorough review of existing regulatory frameworks on external lighting and light pollution in the context of environmental protection and bats, and a review of scientific research papers, reports, and realised case studies on satellite remote sensing technologies. Additionally, personal email correspondence was established with a bat specialist, as well as remote sensing specialists, to provide expertise in the observation of the researched area.

### 2.1. Study Area—An Overview

**Figure 1:**
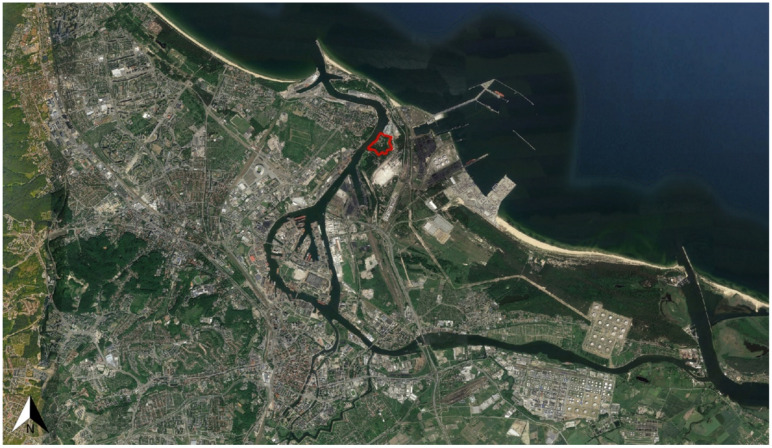
General overview map of the location of the studied area of the Natura 2000 Wisłoujście Fortress site in Gdansk, Poland. Scale: NTS. Source: Google Earth.

**Figure 2:**
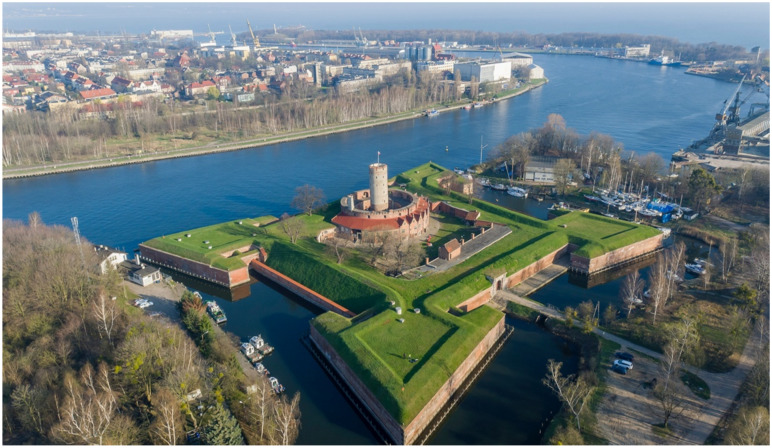
Aerial view of the Wisłoujście Fortress historic complex, part of the Natura 2000 site, located by an old estuary of the river Vistula, which flows into the Bay of Gdańsk. Source: Dariusz Kula/ Museum of Gdańsk.

The main study area (Figure 1) represents the Natura 2000 Wisłoujście Fortress complex (54°23′41″ N 18°40′51″ E) [34]. This historic structure is located in Gdańsk, Poland, by an old estuary of the river Vistula, which flows into the Bay of Gdańsk (Figure 2). It was erected at the end of the 15th century to secure the entrance to the port of Gdańsk from the sea, and continual extensions and modifications were made until the second half of the 19th century. Built from brick and earth fortifications and surrounded by old trees and moats filled with water, it is recognised as the only historic maritime defence complex on the entire coast of the Baltic Sea. The main architectural feature Fort Carré is a square-shaped brick fort with a tower and four domed bastions at the corners. It is also one of the largest wintering grounds for bats in the Pomeranian Voivodeship, with canals and moats adjacent to the fortress providing optimal foraging grounds. For many years, this site, including the fortress complex, has also been the hibernation ground for many rare bats, including the pond bat (*Myotis dasycneme*) which is on the International Union for Conservation of Nature Red List of Threatened Species (IUCN) [35]. In 2004, due to the regular presence of this rare bat (from September to March), this area was entered into the European ecological network “Natura 2000” as a Special Protection Area [36].

Natura 2000 is a complex of protected crucial nesting and breeding sites for threatened and rare species, as well as rare natural habitats. This network extends across all 27 EU countries, on both land and sea [37]. In 2018, the Wisłoujście Fortress complex was also proclaimed to be a Historic Monument—an object of the cultural heritage of Poland [38,39]—as one of the largest preserved, most outstanding works of military architecture, unique in terms of its location, spatial layout, and applied architectural and technical solutions. 

### 2.2. Study Area—Events On-Site

It was not until 1992 that the Wisłoujście Fortress was opened to tourists after renovation works were partially completed on the damaged historical complex. Currently, the fortress is open to visitors from the beginning of May through to the end of September, although there are plans to soon open the venue throughout the rest of the year. Exhibitions that display the history of the monument and the daily life of its former inhabitants are organised here, under the aegis of the Museum of Gdańsk. Various groups provide the reconstruction of historical events where visitors can meet medieval knights, Gdańsk privateers, and soldiers from Gdańsk formations. They can also witness battles re-enacted from various historic wars. 

Between 2011 and 2016, events were limited to the daytime. This included the staging of mock naval battles on the river. These activities involved participants from many countries, who waged a re-enactment of battle on the river in galleons, and the roar of cannons could be heard from a distance. Then, for the first time in history, in 2016, a night spectacle was proposed for the site that incorporated the use of artificial light (video mapping, lasers, fireworks, and pyrotechnics) as well as sound [40]. 

#### Wisłoujście Festival

**Figure 3:**
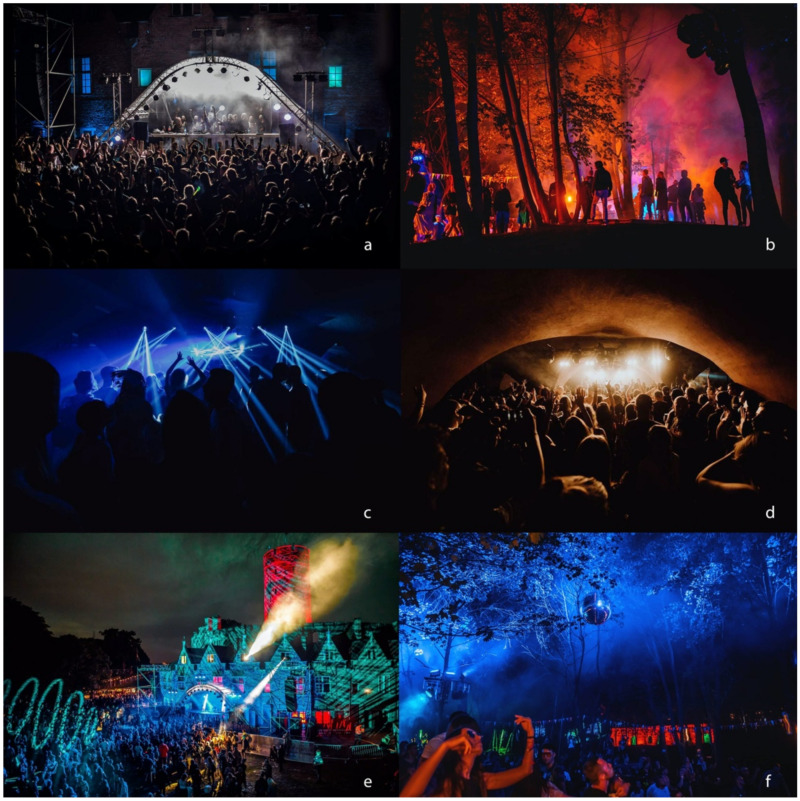
Images from recent events of the Wisłoujście Festival showcasing the extent of ALAN on Nature 2000 site. Source: (**a**) Wojtek Rojek, (**b**–**f**) Zuzanna Sosnowska.

Since 2018, each year the Nature 2000 site is also the location for an outdoor annual summer electronic dance music (EDM) festival, called the Wisłoujście Festival, which is typically held twice in the month of August, over three consecutive evenings [41,42,43,44]. During the summer, the site and fortress are open to the public from 9 am to 6 pm CET, but during the festival period, the opening hours are extended into the early morning hours. Additionally, there are illuminated outdoor stages on-site and one indoor stage (Figure 3) in the Kazamaty area, which is positioned exactly on the site of the bat’s hibernation site. This music festival entertains hundreds of people, all of whom might provide a potential source of disturbance to bats (Figure 3), as existing research from other countries already provides data on the adverse effects of human disturbance on bats for autumn shelters and their hibernacula [45]. 

### 2.3. Pond Bat (Myotis Dasycneme)—General Information

**Figure 4:**
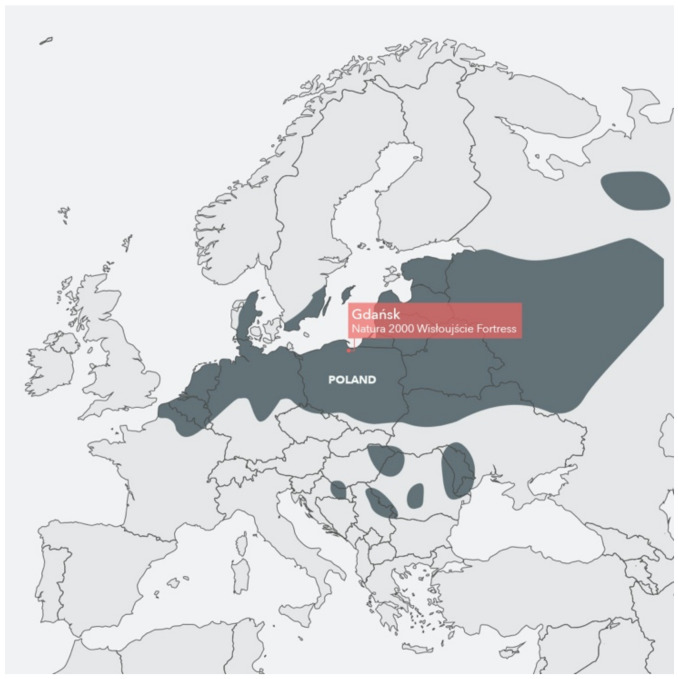
Distribution of the pond bat (*Myotis dasycneme*) in Europe, author’s elaboration based on [49].

This near-threatened bat ranks among the rarest bats in Europe [46]. The distribution of the pond bat (*Myotis dasycneme*) ranges from northern France through Belgium, Netherlands, Denmark, southern Sweden, along northern Germany, Poland, and along the Baltic States to Russia. The southern distribution reaches northern Croatia and Romania (Figure 4). The pond bat is considered to be a medium-range migrant from summer to winter roosts, covering about 100 km. Waterways (canals, rivers) and other water bodies such as lakes are vital places for pond bats to feed as they typically forage on arthropods (mosquitoes, moths, and flies) over calm water. Their calls of echolocation are typically 4–8 milliseconds long with frequency-modulated signals between 25 and 85 kHz [35]. Therefore, any noise from human activity can reduce their hunting [47,48].

While preparing for the winter state of hibernation, pond bats in Poland use underground shelters in the form of fortifications, small cellars, and caves [50], with sporadic hibernation in mines. Bats feed intensively before this time in order to accumulate the fat reserves necessary to survive the winter. Any anthropogenic disruption to this pattern can have extensive negative consequences, e.g., threatening the reproductive and survival rates of individuals, which then harms the colony. In certain countries such as Poland, it is forbidden to photograph, film, or observe these species because this may scare or disturb them [51,52].

Although there are few studies regarding the impact of ALAN on pond bats, in the available literature that does exist, combined with the observations of the genera of *Myotis* bats, ALAN has been recognised as having an adverse effect on their commuting, forging, drinking, and hibernacula [53]. Pond bats are one of the most light-sensitive bats, hence their aversion to artificial lighting. They emerge later from their roosting sites compared to other bats, with sunset time and the onset of darkness being two significant indicators. Current research suggests this behaviour called “*light sampling*”, when the bats test if the levels of light are low enough by undertaking short flights and returning to their roosts, is related to the increased risk of predation [54] as these bats feed over open water away from protective land and tree cover. As the presence of insects over water bodies has been shown to decline gradually after the sun sets [55], the time available to the bats for feeding during the night is crucial. (For more information on bats and ALAN, see Appendix A.) 

### 2.4. Free-of-Charge Satellite Remote Sensing Technologies for ALAN Analysis for Non-GIS and -SRS Experts

Satellite remote sensing is the process of detecting and monitoring the physical characteristics of an area by measuring its reflected and emitted radiation via a distant satellite. This is performed by special measuring tools that collect remotely sensed images.

This method can be used to quantify the upwards radiance on relatively large spatial scales (>the size of one sensor pixel) that would otherwise be difficult to measure on the ground. An upwards radiance is often used as a proxy for the amount of light on the ground in a region and/or the illuminance levels on the ground, although how upwards radiance connects to ground illuminance is still not well understood. 

The ALAN observations available in recent years by the means of data from satellites can be a highly useful tool to gather and analyse information about various aspects [56]. This includes the light pollution caused by human activities and urbanisation, both of which potentially impact the environment in negative ways. Up until 2011, meteorological satellites could record the sensing of night lights as they were equipped with calibrated measuring devices to capture images of the Earth’s visible spectrum of ALAN; however, their dynamic range was relatively low. Sensors aboard recent missions have improved the dynamic range, are more sensitive to faint light, and thus can provide images with much greater accuracy, but the same absolute calibration problems are still present with the Visible Infrared Imaging Radiometer Suite sensor on board the weather satellite Suomi National Polar-orbiting Partnership (in short Suomi NPP-VIIRS). 

While there are only a few satellites/sensors available today that provide constant imaging information for the evaluation of the brightness of the earth’s surface in a given location observed at night from space, some of them require paid access, as indicated in [57]. The only civilian remote sensing platform capable of providing calibrated radiance data of night lights with global coverage right now is VIIRS, and its data are freely available to the public.

However, the information provided from instruments on board two US-based satellites—the Operational Linescan System sensor on board the Defence Meteorological Satellite Program weather satellites (in short DMSP-OLS) and VIIRS—is available free of charge. Since 2016, DMSP no longer supplies night-light data; thus, as a data source, it is only useful for historical comparisons.

The information provided by these two satellites is not user-friendly for non-GIS and -SRS experts and requires specific knowledge and skills to handle the remote sensing geospatial data in order to create an annual, general, and detailed daily overview of light emissions in a given space base of exact coordinates.

A solution to this problem to visually show if general light pollution levels are increasing, constant, or decreasing over time could be interactive web mapping services such as the (1) Light Pollution Map (LPM) and (2) Radiance Light Trends (RLT), completed with NASA’s Black Marble product for detailed evaluation. (For more information, see Appendix C and Table A3 and Table A4.)

## 3. Research Questions

In order to achieve the research objectives and examine the current situation on-site in regards to ALAN and its possible impact on bats, the following research questions were posed:

• Question 1 (Q1). Is the nature of the data presented here capable of distinguishing ALAN as the proximate cause of the population fluctuations and observed decline of the endangered pond bat (*Myotis dasycneme*) in the Natura 2000 area in recent years, as opposed to other influences such as noise or factors unrelated to events like the EDM festival?
• Question 2 (Q2). What are the past and current light pollution levels on-site?
  Was there an identifiable impact from the COVID-19 lockdown on light pollution levels on-site in 2020?
• Question 3 (Q3). In recent years, a new tool in the form of remote sensing has been applied to assess the scale of light pollution. Is remote sensing data also appropriate to research the Natura 2000 site, located in the Wisłoujście Fortress?
• Question 4 (Q4). Are the legal conditions for the protection of the Natura 2000 Wisłoujście Fortress sufficient?

## 4. Materials and Methods

This part presents a broad report and clarifies the procedure of data collection and content analysis to test the research questions.

### 4.1. Data Collection for the Analysis of Land Cover in the Natura 2000 Wisłoujście Fortress

#### 4.1.1. Source

Reference data from the Database of Topographic Objects (BDOT10k) were downloaded from the geoportal.gov.pl (accessed on 5 May 2021) portal [58]. (This web portal provided by the Head Office of Geodesy and Cartography in Poland is used to find and assess geographic information and associated geographical services via the Internet.)

BDOT10k [59] is a spatial database with details corresponding to a 1:10,000 topographic map with public records (as a part of the Polish National Geodetic and Cartographic Resource). These data allow for extensive analysis.

#### 4.1.2. Analysis Methods

The data were processed using a free and open-source cross-platform desktop geographical information system application QGIS version 3.16. Vector layers were imported from the BDOT10k database and saved as shapefile files. This procedure allowed data editing, processing, and analysis with all the tools available for vector layers. The data on the type of land cover (defined in BDOT10k as level 1, of the PT code) were combined into one layer, while the objects retained all their attributes. 

This allowed for the intersection of a new, single layer (not a group) with the layer defining the polygon for the Natura 2000 Wisłoujście area. After such a procedure, it was possible to analyse each of the objects—calculate its area and then group the objects into groups according to levels 2 and 3 of the objects of the BDOT10k base.

### 4.2. Data Collection for the Analysis of Changes in the Number of Pond Bats on the Natura 2000 Wisłoujście Fortress Site

Written communication (email correspondence) with bat specialists was performed to obtain an official report with data about the autumn swarming season count of the pond bat for the period between 2011 and 2020 in the bastions of Fort Carré of the Wisłoujście Fortress. The calculations of mean and population standard deviations were performed as a method for quantitative data collection and analysis.

### 4.3. Data Collection for the Analysis of Daytime and Nighttime Events Held at the Natura 2000 Wisłoujście Fortress Site

An internet-based research method [60] was used to identify all daytime and nighttime events connected to the Wisłoujście Fortress. 

The search began in 2011 (when the first autumn bat count took place) to the year 2020 and was narrowed down to the summer months of May through to September when the complex is open to the public.

All data were collected in a process that combined various search strategies. This included online articles in a local newspaper, information on the Museum of Gdańsk website, and videos on its YouTube channel, as well as news from local TV stations and the regional internet portal Trojmiasto.pl. The respective events were identified by a typical keyword-based search method using the words and phrases in the Polish language such as: “Obrona Twierdzy Wisłoujście”, “Bitwa morska Twierdza Wisłoujście”, “Bitwa Morska i Lądowa Wisłoujście 1577”, “Twierdza Wisłoujście spektakl świateł”, or “Festiwal Wisłoujście”.

### 4.4. General Overview of Changes in Light Pollution Levels 

#### 4.4.1. Source

For the purpose of providing a general overview of changes in light pollution levels on the research site, four criteria were identified including: (1) the service should be available free of charge; (2) data should be available from 2012 (when VIIRS-DNB data first became available) to the present day; (3) any obtained spatial resolution of data needs to be higher than that provided by DMSP satellites; and (4) both daily and annual datasets should be available.

Out of seven available satellites/sensors, the information gathered by the National Oceanographic and Atmospheric Administration (NOAA) in America, from the weather satellite Suomi National Polar-orbiting Partnership (SNPP) Visible Infrared Imaging Radiometer Suite (VIIRS), seemed to be the most appropriate because it was the only data source obtained from this satellite that fulfilled all four criteria set above.

An analysis was performed based on the data provided by the Light Pollution Map (LPM) interactive web application which uses information from Suomi NPP that includes the Day/Night Band of the Visible Infrared Imaging Radiometer Suite instrument (VIIRS-DNB). As there was an issue with VIIRS-DNB data in dark places (connection to the temporal variation of natural light sources such as airglow limited the ability of night light sensors to detect changes in small sources of artificial light), the authors decided to use the Radiance Light Trends (RLT) web service for comparison, which can correct this problem by applying VIIRS-DNB (zero-point calibration) [61]. 

#### 4.4.2. Analysis Methods

**Figure 5:**
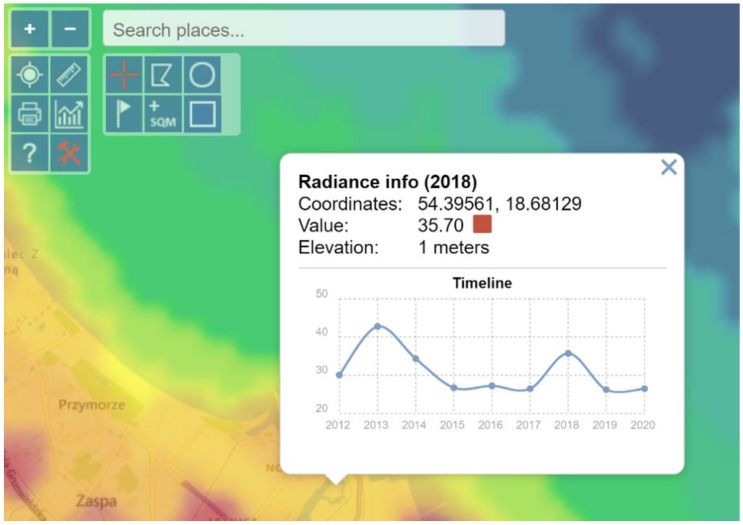
An example of radiance levels based on a selected year and point within the Wisłoujście Fortress area, with the help of the “Point information tool” from the LPM web portal. Source: authors’ elaboration based on the graphics displayed by the LPM service.

In the first stage, a visual analysis was performed on light pollution maps for the years 2012–2020 in the area of the Wisłoujście Fortress and surrounding areas, based on the LPM service. A composition was made of screenshots taken in the same terrain location. This composition included maps for individual years. The next step was to analyse the data provided by the web portal. For this, an extended tool from the “Toggle extended toolbar”—“Point information” group was used. Figure 5 shows the available options. After moving the cursor over the graph, individual radiance values for a given year were read in the indicated place. The data that were interpreted in this way were tabulated for 7 points (point 1—Wisłoujście Fortress, points 2–6—the surrounding areas). Table 1 shows the coordinates of the analysed points.

In order to verify the averaged situation regarding the light pollution that impacts the Natura 2000 Wisłoujście Fortress site, an analysis of radiance changes during 2012–2020 was performed in areas indicated by a circle with a radius of 1 km, 2 km, and 3 km, with the Wisłoujście Fortress at its centre. The results were also tabulated and are commented on in Section 5 of this article. For this purpose, the “Area (circle) statistics and data export” tool on the LPM web portal was used.

Additionally, relevant data provided by the second service were compared using https://lighttrends.lightpollutionmap.info/ (accessed on 5 May 2021). For this part of the analysis, the “Analyse data by selecting pixel” tool was used. After clicking on the selected place, a graph of radiance data was generated for the period 2014–2020 for the selected point. The collected data were compared for the indicated 7 points.

### 4.5. Detailed Changes in Light Pollution 

#### 4.5.1. Source

In order to obtain more detailed information, NASA’s Black Marble service with daily calibrated, corrected, and validated data—the VNP46A2 Daily Moonlight-adjusted Nighttime Lights (NTL)—was used [62]. This generates seven datasets:

1. DNB BRDF-Corrected NTL
2. Gap-Filled DNB BRDF-Corrected NTL
3. DNB Lunar Irradiance
4. Latest High-Quality Retrieval
5. Mandatory Quality Flag
6. Cloud Mask Quality Flag
7. Snow Flag

For the purposes of this article, the data for the area of Gdańsk were analysed. These were data for the h19.v03 position. (These data specify a “tile” raster from all available artwork—more specifically, the coordinates of the entire artwork.)

#### 4.5.2. Analysis Methods

Data to be analysed must be converted from hdf to geotiff format. This allows analysis with the use of programs such as QGIS or MATLAB, which were used for this research. The file conversion was performed with the Python console using QGIS3.16 software. Each dataset was split and saved as a separate geotiff. The entire procedure was recommended on the following website https://blackmarble.gsfc.nasa.gov/Tools.html (accessed on 5 May 2021).

Two areas were analysed with the help of the image processing and matrix analysis tool available in MATLAB 2020b. The first was the area of 9 pixels which coincided with the Wisłoujście Fortress. The second was an area with 1296 analysed pixels located deeper into Gdańsk (south-west).

On the basis of these obtained matrices, several attempts of analysis were carried out, which assumed the analysis of various time periods and various types of data. (A detailed description and results are outlined in Section 5 Results.)

### 4.6. International, European, and Polish Regulatory Frameworks—The Most Important Approaches for Environmental Protection of Bats in the Context of Ecological Light Pollution 

#### 4.6.1. Analysis of Lighting Regulatory Frameworks (Soft Law Protection)

This examination involved an in-depth assessment of published International and European lighting guidelines, procedures, standards, and codes. 

#### 4.6.2. Analysis of Legal Conditions for the Protection of the Natura 2000 Wisłoujście Fortress (Hard Law Protection)

The basic research method used in the study of legal conditions for the protection of Natura 2000 sites is a dogmatic analysis of normative material, where the subject of the study is the content of existing legal norms and the interpretation of the current law with an evaluation of its application, as well as formulation of de lege ferenda conclusions [63]. Legal-dogmatic research concerns researching current positive law and de lege ferenda conclusions that apply to possible future amendments to existing legislation.

The problem was presented from the perspective of European Union law (Birds Directive and Habitats Directive) and Polish law regulations, which implement EU standards concerning the organisation and function of Natura 2000 sites in the territory of Member States. The interpretation of national law includes: the Nature Conservation Act; the Act on the disclosure of information about the environment and its protection, public participation in environmental protection, and environmental impact assessments; and the Environmental Protection Law and implementing acts (ordinances). Of key importance was also the analysis of the local law act, i.e., the Order of the Regional Director for Environmental Protection in Gdańsk, which sets out the protection management plan for the Natura 2000 Wisłoujście Fortress site.

## 5. Results

### 5.1. Assessment of the Natura 2000 Wisłoujście Fortress, Together with an Assessment of Land Cover in this Area

**Figure 6:**
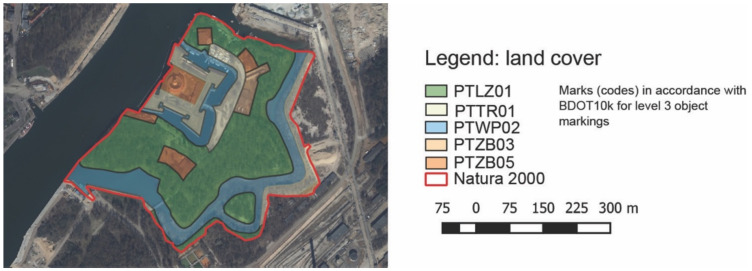
Visual analysis of types of land cover within the Natura 2000 Wisłoujście Fortress, based on BDOT10k. Source: authors’ elaboration.

The area of the Wisłoujście Fortress is entirely covered by Natura 2000, which encompasses up to 16.17 ha (Figure 6). This area consists of: flowing surface waters, built-up areas, forests, and grassy vegetation which are presented in Table 2 in more detail.

The area is mostly covered with forest and wooded areas, i.e., 47%, then with surface water, i.e., 27%, and grassy vegetation, i.e., 16%, and built-up areas account for 10%. It can be determined that the natural environment (water and vegetation) significantly exceeds the analysed area of the Natura 2000 Wisłoujście Fortress (Figure 6).

### 5.2. Analysis of Changes in Light Pollution Levels and the Number of Captures of the Pond Bat during the Annual Autumn Swarming Season

**Figure 7:**
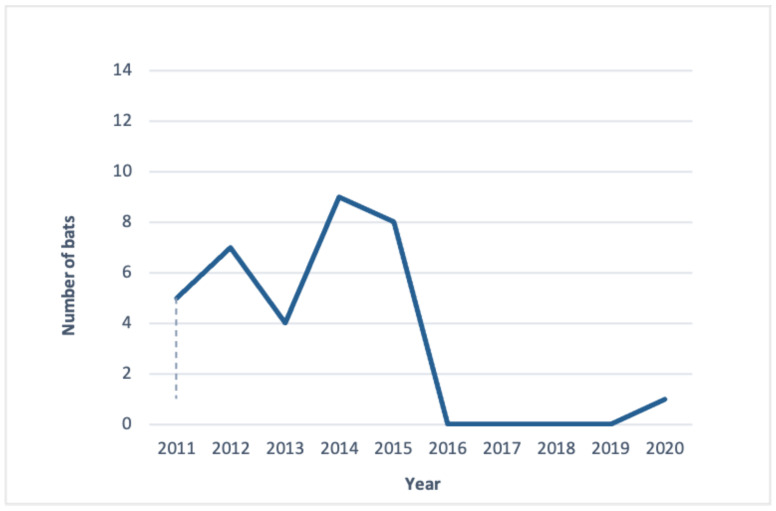
Changes in the number of captures of the pond bat during the autumn swarming season count in the bastions of Fort Carré of the Wisłoujście Fortress in October between 2011 and 2020, indicating a reduction in their numbers to zero, starting from 2016.

Since 2011, bat monitoring has been performed annually on-site. The autumn swarming count has taken place in October, but since 2017, it has also been conducted in mid-September due to the total decrease of bat numbers starting in the year 2016, as observed and recorded by bat specialists. This count typically starts at 6 pm and concludes by 7 am. The bat data for this research were based on information provided by a chiropterologist’s annual studies performed for the Regional Directorate for Environmental Protection in Gdańsk (Figure 7).

Introducing a nighttime event with exterior illumination (Table 3) might adversely affect the natural environment and cause a disruption in the local ecosystem, impacting the population of bats. In order to provide evidence for this claim, we used a standard deviation—a typical statistical tool that is applied to find out how far from the mean a typical value from a distribution can be.

We calculated the mean of the pond bat population for the first five years of our observation (before the nighttime events were introduced), which was 6.6 with a standard population deviation of 1.85. After the nighttime events were introduced over a period of the next five years, the mean for the bat population changed to 3.46 being measured for the period of ten consecutive years since starting the experiment. The respective standard deviation almost doubled in value to 3.46. This suggests that the growing fluctuations in the pond bat population are most likely due to a new factor (nighttime events) being introduced in the local ecosystem.

### 5.3. Analysis of Changes in Light Pollution Levels

#### 5.3.1. General Overview of Annual Changes in Light Pollution

Annual changes in radiance values at night are very important when considering the protection of bats and the annual changes in their population. The lighting situation in Gdańsk has changed over the last decade. This effect is also visible in the surrounding land of the Wisłoujście Fortress and the fortress itself.

**Figure 8:**
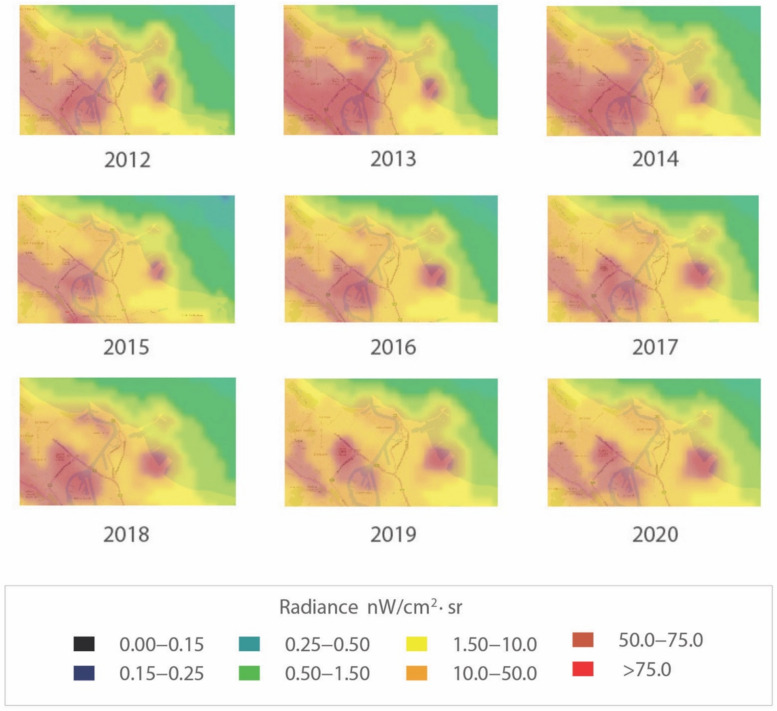
The composition of light pollution maps for the years 2012–2020 for the Wisłoujście Fortress Natura 2000 site in Gdańsk and the areas adjacent to it. Source: authors’ elaboration based on the graphics displayed by the LPM service.

The overall visual analysis of the area presented in Figure 8 (about 6 × 9 km) shows there was a change in light pollution levels in 2012–2014, indicated by the increased intensity of the red colour (radiance level > 75 nW/cm^2^·sr). The year 2015 was characterised by the lowest red colour intensity. (We believe the reason for this change is based on Gdańsk participating in a program to upgrade its street luminaires [65,66] to luminaires with different lamp heads to reduce upwards light spill, as well as a change in light sources from low- and high-pressure sodium lighting to metal halide and later LED lighting. See Section 7 Limitations of the Study for more information.) Meanwhile, the following years until 2019 show ALAN has increased and polluted the analysed areas to a greater extent due to the expansion of residential districts, the revitalisation of Letnica—a district of Gdańsk city—the establishment of new companies, and the expansion of older ones in this area. It can also be seen that between 2017 and 2020, the areas that were sources of this pollution could be isolated on the maps (Table 1). In 2019, there seems to be a visible reduction in levels compared to 2018 due to possible cloud cover (in Gdańsk, seasonal variations over the course of the year affect the average percentage of the sky covered by clouds), and there was also some decrease in light pollution due to the decline in human activity from lockdown in 2020, e.g., in the Northern Port area.

**Figure 9:**
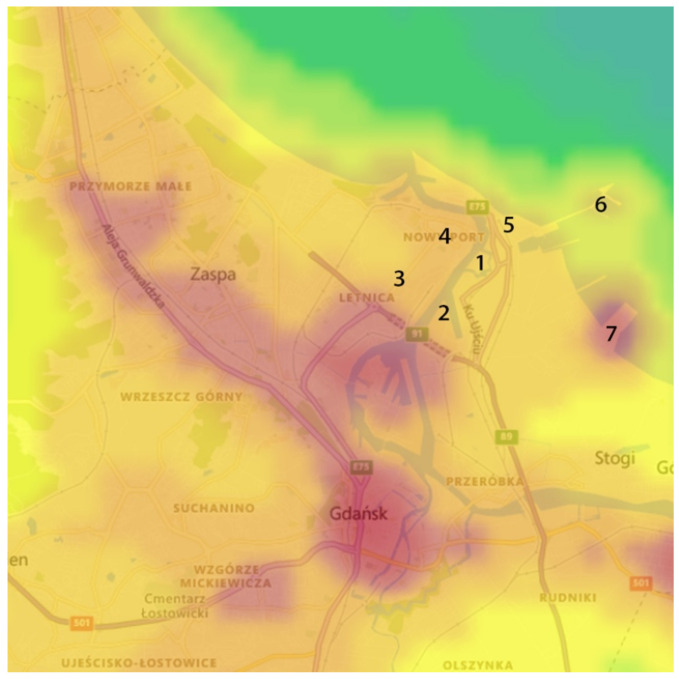
Locations of seven points for light pollution level analysis. Source: authors’ elaboration based on the graphics displayed by the LPM service.

Due to these dynamic changes in light pollution levels, we decided to present the values of light pollution in individual places as they could be identified by the skyglow emanating from them—and these individual places directly impact the Natura 2000 site (Figure 9). 

**Figure 10:**
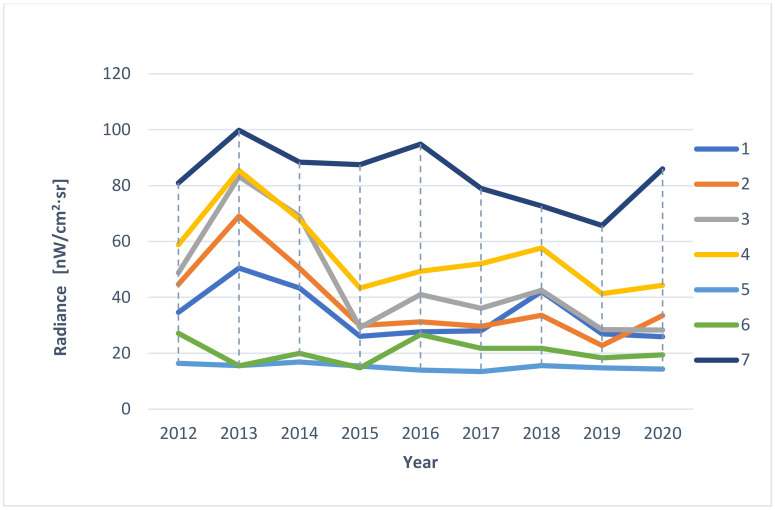
Radiance changes in 2012–2020 according to the service https://www.lightpollutionmap.info/ (accessed on 5 May 2021).

**Figure 11:**
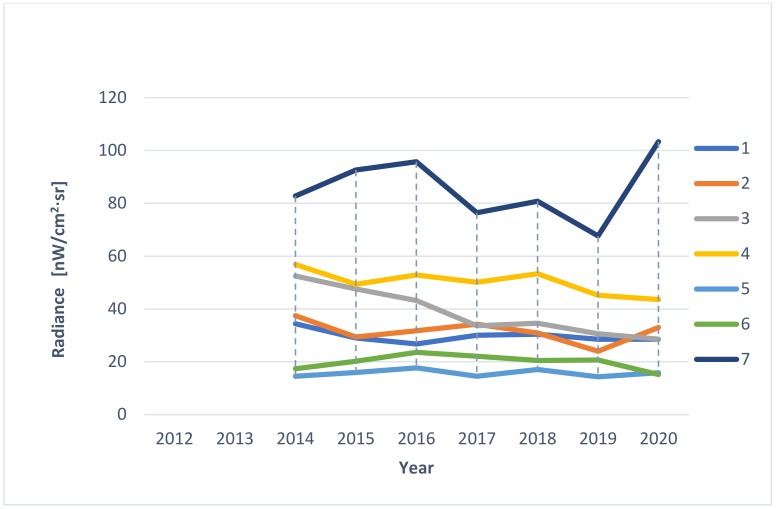
Radiance changes in 2014–2020 according to the service https://lighttrends.lightpollutionmap.info/ (accessed on 5 May 2021).

The results of the analysis for individual points are presented in graphical form. Based on information from services such as: https://www.lightpollutionmap.info/ (accessed on 5 May 2021) (Figure 10) and https://lighttrends.lightpollutionmap.info/ (accessed on 5 May 2021) (Figure 11).

**Figure 12:**
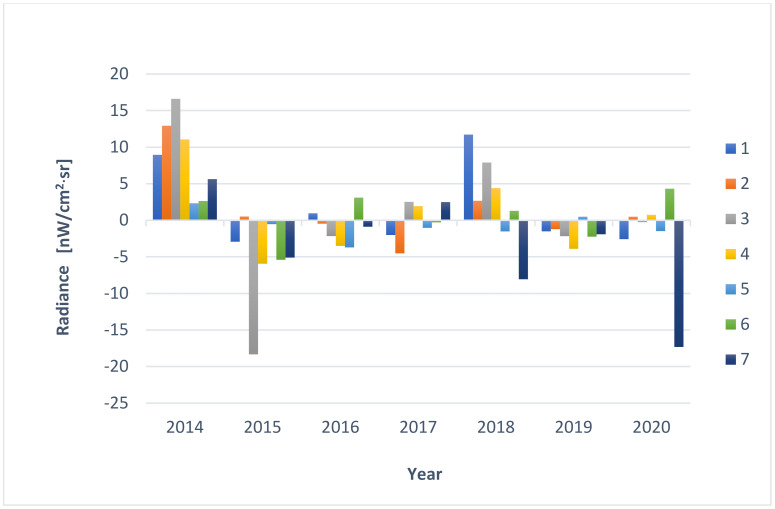
Differences in radiance values in 2014–2020 determined by two services. Source: authors’ elaboration.

It was noticed that the data provided by both services are different. The differences for individual points (1–7) in the years are presented in Figure 12. The service https://lighttrends.lightpollutionmap.info/ (accessed on 5 May 2021) does not provide data from the year 2012 and 2013; therefore, for these years, a comparison was not possible. In the presented chart, it can be seen that the years 2014, 2015, and 2018 are the years where the differences in the data are substantial (they stand out from other years, as well as for a significant part of the measurement points, they exceed the value of the difference of +/− 5 nW/cm^2^·sr). For a more detailed analysis, the authors decided to additionally include Table 4, which presents the exact values along with the determined average, minimum, and maximum values (for annual values and for measurement values for individual points). The data in the table confirm the results discussed before that the years 2014, 2015, and 2018 are characterised by the highest mean differences (respectively, 8.59, 5.54, and 5.37 nW/cm^2^·sr). Additionally, it was noticed that the values of the differences are higher for pixels where the level of radiance is also high—points: 3, 4, and 7.

The authors wish to draw attention to the use of easily available LP data analysis tools but also to emphasize their limitations. This means obtaining results at a different level requires these services to be used with an awareness that there may be possible errors. Nevertheless, the authors also emphasize the advantage of RLT due to the zero-point calibration (see Appendix C for more information).

**Figure 13:**
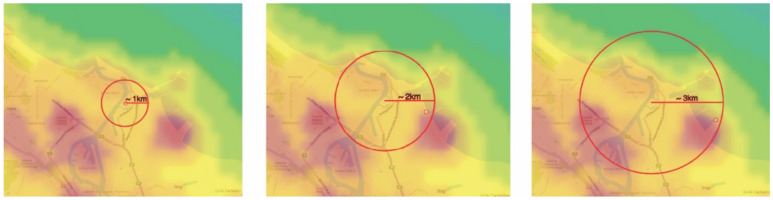
Area enclosed by a circle with a 1 km, 2 km, and 3 km radius. Source: authors’ elaboration based on the graphics displayed by the LPM service.

**Figure 14:**
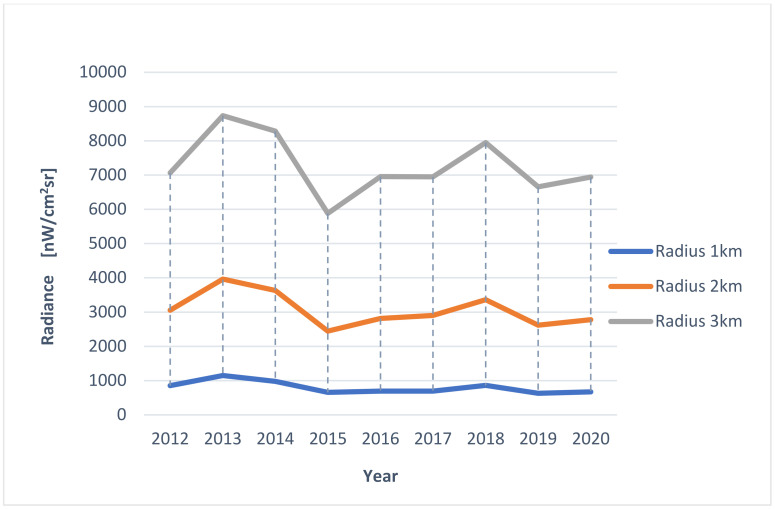
Light pollution changes from 2012 to 2020 based on a 1 km, 2 km, and 3 km radius. Source: authors’ elaboration based on data from https://www.lightpollutionmap.info/ (accessed on 5 May 2021).

Another way to present the light pollution situation is the analysis of the Natura 2000 site enclosed by a circle with a 1 km, 2 km, and 3 km radius, showing changes (increases/decreases) for individual years, correlated for each of the areas (Figure 13 and Figure 14). This analysis suggests that light pollution levels generated inside a 3 km radius and beyond might have a negative impact on the Natura 2000 site.

In Figure 14, there are what appear to be unusually high radiances in 2013 and 2018 that are attributable to anomalously high values in the winters of those years, which affect one month out of twelve (as revealed in the RLT analysis based on monthly cloud-free composite imagery). Therefore, the value of this approach is limited given that it does not account for the influences that change radiances on different timescales (nightly, monthly, annually). Accounting for these outliers, there is little variation in any of these circles during the study period.

#### 5.3.2. Detailed Overview of Daily Changes in Light Pollution

An attempt was also made to analyse the data recorded on specific days during the periods of cultural events organised at night in the Wisłoujście Fortress, which could affect the brightness of the area and the sky on a given night. VNP46A2 data were collected for the area of interest. The dates and information about the data are summarised in Table 5. Due to heavy cloud cover, poor data quality, or the lack of coverage by the measurement of the area on a given date, the analysis for each event was not possible.

**Figure 15:**
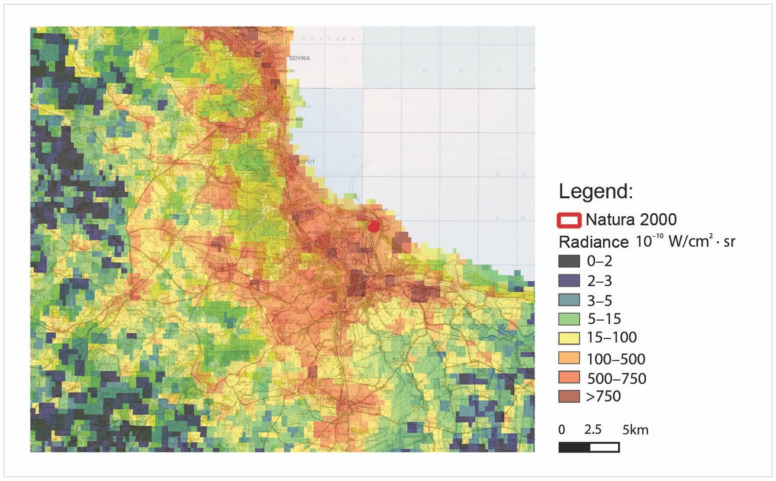
Radiance map taken on 28 August 2020 showing Gdańsk and its surroundings.

**Figure 16:**
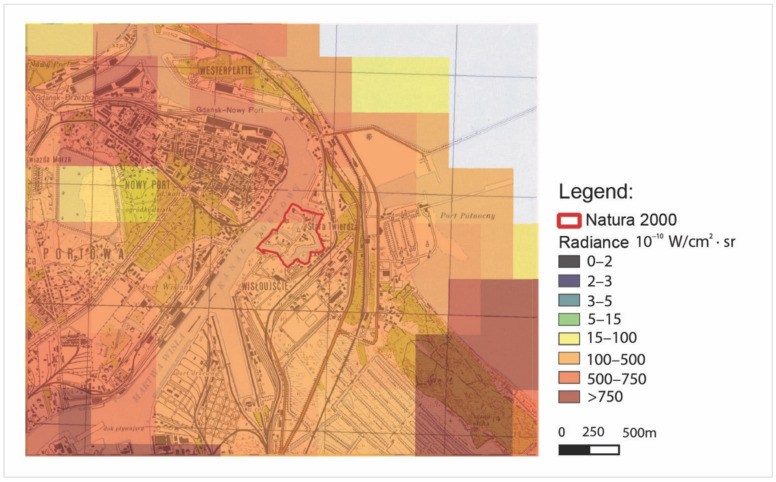
Radiance map taken on 28 August 2020 showing a close-up of the Natura 2000 Wisłoujście Fortress.

Figure 15 and Figure 16 represent radiance maps taken on 28 August 2020 in two different scales of spatial mapping obtained as the raster product of the composition of VNP46A2 of the data DNB BRDF-Corrected NTL raw sources. The images represent a different area on this scale. f15 is the area of the Tri-City, and f16 is the area of the Wisłoujście Fortress with its immediate vicinity.

The grey colour of pixels represents the lowest radiance levels between 0 and 0.2 nW/cm^2^·sr = 0–2 10^−10^ W/cm^2^·sr, while the red colour represents the highest, brightest radiance levels of >75 nW/cm^2^·sr = >750 10^−10^ W/cm^2^·sr.

Nevertheless, the analysis with the images presented in this way allows for a preliminary interpretation of the situation—pinpointing the potential directions of sources of light pollution on a certain specific day. For the authors, it was crucial to clarify and verify these measurements as they relate specifically to the Wisłoujście Fortress. The larger-scale area of the graphics (Figure 15) demonstrates that the fortress area is covered with four pixels. From this figure, it is very difficult to identify any light pollution levels of the Natura 2000 Wisłoujście Fortress site due to its scale. (In Figure 16, the area obtained approximate radiance levels of 10–75 nW/cm^2^·sr = 100–750 10^−10^ W/cm^2^·sr).

As this information is rather unspecific, it only provides an approximation with a large range of radiance (information for one night). It was therefore decided to analyse the obtained information based on the composition of the VNP46A2 of the data DNB BRDF-Corrected NTL in a bar graph form below. The authors analysed the Festival Wisłoujście event that took place on 28–30/08/2020 (see Table 5).

**Figure 17:**
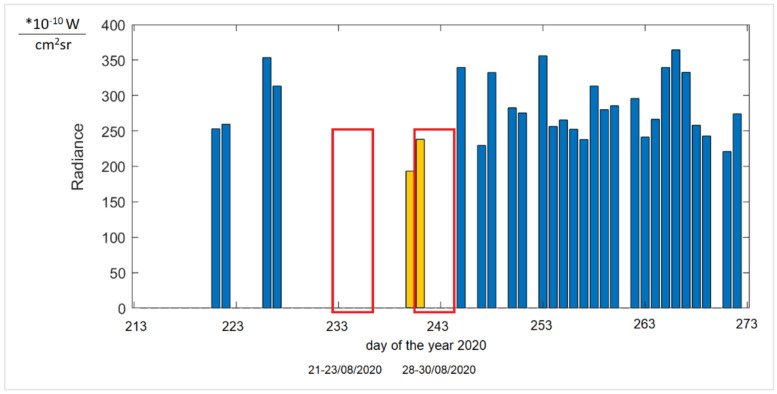
Radiance levels at night from registration August–September 2020 for the area of the Wisłoujście Fortress. (The red rectangles were used to mark the period in which the festival took place. The colour yellow indicates the availability of high-quality data which could be used for the analysis. The blue colour indicates availability of high-quality data, except on festival nights. Zero values for individual days mean that the data recorded for that area were either unavailable or of poor quality). * Unit of radiance.

The graph in Figure 17 presents the average value of radiance for the area of the Wisłoujście Fortress, represented by nine pixels, for the period of August and September 2020. The reason for the analysis of a longer period (before and after the festival) was the need to reference the recorded values to the situation before and after the event.

**Figure 18:**
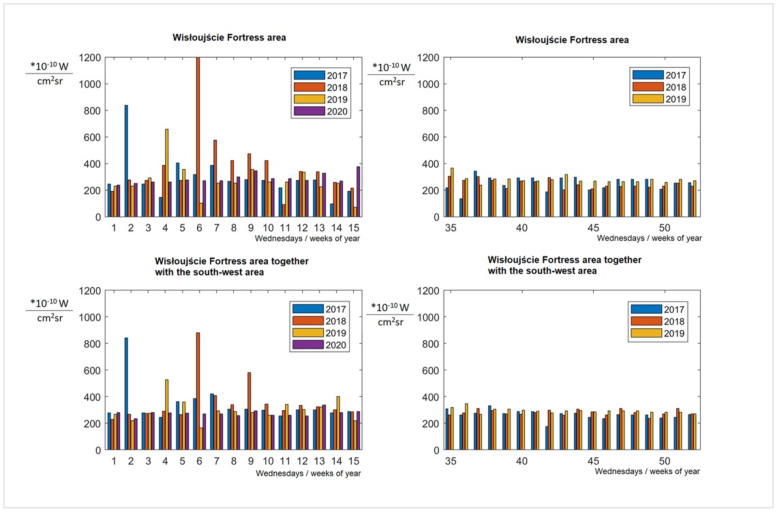
Graph of the radiance value for individual Wednesdays/weeks of the 2017–2020 period in the analysed areas. Source: authors’ elaboration. * Unit of radiance.

Based on the graphical evaluation, it can be observed that, during the Wisłoujście festival, the radiance value did not differ from other recorded days in August and September. The day before the festival, it was lower than on the day of the festival. However, taking into account the overall data, it cannot be confirmed that the sensor actually recorded light phenomena during the music concert. There might be several reasons for this as follows: (1) the time of registration (it is possible that at the time of registration the lights were turned off), (2) the registration angle (at the time of registration other objects could have covered the light sources (e.g., some lights were covered by tree foliage), (3) the spectral range of the sensor (the lights emitted during the festival could have emitted electromagnetic waves beyond the spectral range of the camera sensor), or (4) some other unknown causes (e.g., phenomena occurring in the atmosphere). (These are discussed in more detail in Section 7. Limitations of the Study.) The above results can be seen as indications only, and this is the reason for a more detailed analysis of the period between 2017 and 2020 (Figure 18). It was decided to analyse all available Gap-Filled DNB BRDF-Corrected NTL data for these years for Wednesday to see if there were any variations between the seasons as usually, in the middle of the week, there are no events, etc.

**Figure 19:**
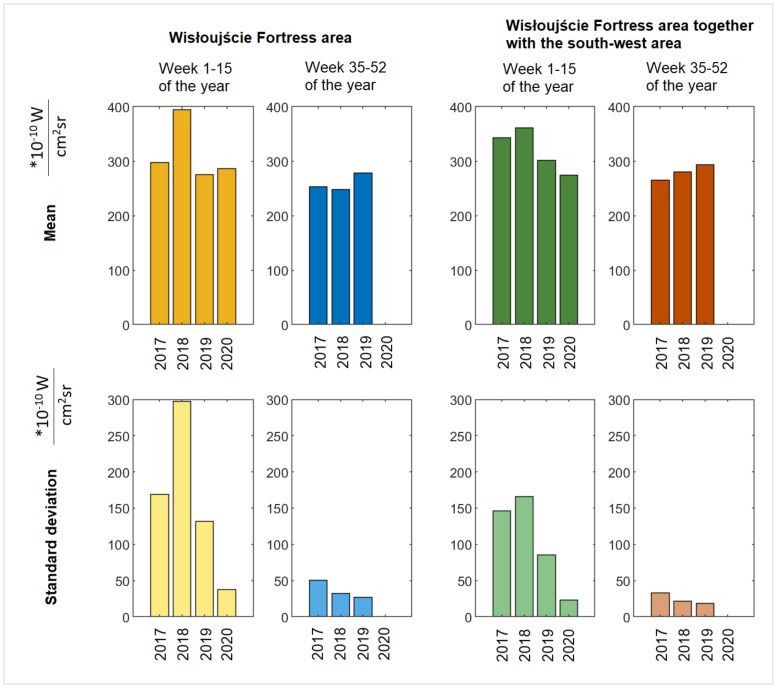
Average and standard deviation of the radiance analysed for the period between 2017 and 2020 for the areas concerned. Source: authors’ elaboration. * Unit of radiance.

For the area of interest, there are no data on the measurement coverage for the period between 2017 and 2019, within the average range from week 16 to 34 (April to mid-August). However, in 2020, additional data are missing (unavailable analysis performed as of 4 March 2021) for the 16th week. Figure 18 shows the obtained values for individual weeks/Wednesdays. The horizontal axis indicates the Wednesday for each of the recorded years. The two upper graphs show the average values for the fortress area itself, the lower ones for the fortress area together with the area adjacent to the south-west side. In order to be able to better understand the data, Figure 19 was prepared, showing the values of annual averages and standard deviations for the data indicated in Figure 18.

From Figure 18 and Figure 19, several conclusions can be drawn: (1) The radiance is more varied in weeks 1–15 (January to mid-April) than in weeks 35–52 (September to December), which is confirmed by the values of the standard deviation. One can reasonably surmise that intermittent snow cover could explain this difference. (2) The year 2018 was a year when significant sudden increases in radiance in the analysed areas were observed; however, for the purposes of a preliminary analysis of light pollution in the Natura 2000 Wisłoujście Fortress site, the obtained results seem to be insufficient to provide an answer to our research question. Our data suggest a link between the festival and the local bat population, but it is inconclusive. (3) The indicated differences require further, more detailed research to determine their actual cause. Therefore, a properly controlled before/during/after study would be helpful. 

### 5.4. Existing Regulatory Frameworks on ALAN in the Context of Environmental Protection and Bats

There has been a number of very useful guidelines created with the help of biologists and bat specialists relating to bats, including information on ALAN and light pollution. However, the identified procedures are inadequate, and they insufficiently address conservation planning in terms of the application of illumination.

In addition, available lighting standards and codes ineffectively cover the levels of ALAN at Natura 2000 sites, as they only relate to street or building lighting. The recommended lighting levels in existing lighting standards (see Appendix B, Table A2) are excessive, especially for natural environments as they have been developed for human perception and photopic vision. (The illuminance thresholds at which bats are known to suffer harm are lower than the recommended illuminances in the standards.) Therefore, the establishment of proper lighting requirements including appropriate light levels and spectral power distributions is of significant importance, including the recommendation that some places such as Natura 2000 be left in naturally dark conditions. (For more information see Appendix B, Table A2.) 

### 5.5. The Regulation of the Natura 2000 Sites in European Union Law: Birds Directive and Habitats Directive

The legal conditions involved with establishing the Natura 2000 sites have their connotations in European Union law. In accordance with the legal definition set out in Article 3(1) of the Habitats Directive, Natura 2000 means “*a coherent European ecological network of special areas of conservation*”. The European Commission clearly defines Natura 2000 as “*a site of core breeding and resting sites for rare and threatened species, and some rare natural habitat types*” and considers it a “*cornerstone of Europe’s legislation on nature conservation*” [67], which “*represents the most ambitious and large-scale initiative ever undertaken to conserve Europe’s natural heritage”* [68].

In essence, this form of conservation aims to create special nature areas where certain animal species and their natural habitats are protected in order to preserve biodiversity. These species and habitats are the common heritage of the European Community; thus the legal framework for qualifying an area for a Natura 2000 site is set out in the following European Union laws:

1. Directive 2009/147/EC on the conservation of wild birds (also known as the “Birds Directive”) [69];
2. Council Directive 92/43/EEC on the conservation of natural habitats and wild fauna and flora (also known as the “*Habitats Directive*”) [70].

These legal acts define two different types of area protection: Special Protection Areas (SPAs) and Special Areas of Conservation (SACs). The first type is an area where protective measures are provided for European bird species and habitats of special interest. In accordance with Article 4(1) of the Birds Directive, “*Member States shall classify in particular, the most suitable territories in number and size as special protection areas for the conservation of these species in the geographical sea and land area where this Directive applies*”. The second type of area is explicitly defined in Article 1(l) of the Habitats Directive as *“a site of Community importance designated by the Member States through a statutory, administrative and/or contractual act where the necessary conservation measures are applied for the maintenance or restoration, at a favourable conservation status, of the natural habitats and/or the populations of the species for which the site is designated”*. Both SPAs and SACs make up the Natura 2000 sites.

All Member States of the European Union are obliged to designate Natura 2000 sites and establish a legal basis for the development of a network of sites that protects endangered species and natural habitats. What should be emphasised is that a directive is a legal act that only sets a goal for all Member States to achieve, without specifying how to implement this. This means that the way to achieve a certain goal is determined by national law. In accordance with the principle of subsidiarity, Member States draw up their own national policies for the implementation of the Habitats and Birds Directives. None of these directives specify the protective measures that Member States should implement. Their choice, however, depends solely on the Member State concerned, and thus the diversity of protection measures in particular countries results from different natural, socio-economic, and legal conditions. 

The European Union is entitled to act in all environmental policy areas, which covers not only air and water pollution, waste management, and climate change but also artificial light pollution. The legal basis for action directly results from Articles 11 and 191–193 of the Treaty on the Functioning of the European Union (TFEU) [71]. The key strategies and plans of the European Union do not address the problem of light pollution. This specific source of pollution is not mentioned at all by the EU Green Deal, which is considered the European Commission’s flagship response to contemporary problems related to progressing climate change and environmental degradation [72]. A core part of the European Green Deal, the Biodiversity Strategy 2030, does not even take light pollution into account [73]. The omission of light pollution makes the European Union policy incomplete, and this requires urgent attention to resolve this matter. Some conscious states of the European Union have introduced national laws aimed at reducing harmful emissions of artificial light. The most recent regulations in this field have been issued by Croatia (Law on protection against light pollution) [74], Germany (Bavarian Nature Conservation Act) [75], and France (Decree on the prevention, reduction, and limitation of light pollution) [76]. 

### 5.6. Legal Conditions of Natura 2000 Site Protection in Poland

In Poland, the basic act that regulates the objectives and principles of protection on Natura 2000 sites is the Nature Conservation Act [77]. The legislative provision considers Natura 2000 sites a special form of nature protection, defined as *“a special bird protection area, a special area of conservation habitats or the area of relevance to the Community, created in order to protect the population of wild birds and natural habitats or species which are of Community interest*”. All three categories of protection areas mentioned in the legal definition form the Natura 2000 network. Moreover, in the light of Polish law, a Natura 2000 site may include part or all of the areas and objects covered by other forms of nature protection, such as: national parks, nature reserves, landscape parks, areas of protected landscape, natural monuments, documentary sites, and ecological sites, as well as nature and landscape complexes. 

The provisions of the Nature Conservation Act set the framework for the protection in each Natura 2000 site, i.e.,:

1. Tasks and competencies of public administration bodies responsible for Natura 2000 sites;
2. Preparation of the Natura 2000 protection management plans;
3. Supervision of Natura 2000 sites;
4. Prohibitions in Natura 2000 sites;
5. Scope of allowed activities in Natura 2000 sites;
6. Consequences of undertaking activities that may have a significant negative impact on the conservation objectives of Natura 2000 sites.

The specific criteria for the classification of areas as Natura 2000 sites, as well as the designation, boundary change, and liquidation of a special bird protection area or special habitat protection area, are regulated by separate ordinances.

All draft policies, strategies, plans, and programmes, as well as planned projects likely to have a significant impact on a Natura 2000 site, require an appropriate impact assessment under the terms of the Act on the disclosure of information about the environment and its protection, public participation in environmental protection, and environmental impact assessments. Unfortunately, the Ordinance of the Council of Ministers on projects likely to significantly affect the environment does not include the emission of artificial light as a factor justifying an environmental impact assessment, even in sensitive sites such as Natura 2000. This is also not directly stated in a provision specifying information for the environmental impact assessment report. Such a legislative oversight appears to be incompatible with Directive 2011/92/EU on the assessment of the effects of certain public and private projects on the environment. Annex IV, points 1(d) and 5(c) of this directive make it clear that this assessment includes an estimate of the expected light emissions of a project and a description of the likely significant effects resulting from light pollution.

For more information on the Natura 2000 Wisłoujście Fortress site PLH220030 and its protection management plan, see Appendix D.

## 6. Discussion

As the impact of ALAN on pond bats remains poorly researched and documented, our understanding of its consequences remains limited.

Therefore, in this paper, we investigated if the artificial illumination of the historic Wisłoujście Fortress in Gdańsk, Poland (part of the Natura 2000 network), during nighttime events, including an outdoor electronic dance music (EDM) festival, might be responsible for increased light pollution and the decline in recent years of the bat species of pond bat (*Myotis dasycneme*).

Moreover, we wanted to see if there was a positive effect from the COVID-19 lockdown on the number of bat species in 2020 (as this was observed in other countries with other organisms), if light pollution levels estimated with satellite-based imagery can be used to help assess its scale in the Natura 2000 Wisłoujście Fortress, and if this tool is appropriate for this undertaking. Lastly, we examined the sufficiency of legal conditions for the protection of the Natura 2000 Wisłoujście Fortress in Poland.

Although we could not identify an increase in light pollution levels on the days when the Wisłoujście Festival took place due to various limitations (see Section 5.3 and Section 7), our research indicates that the overall levels fluctuated over the years on-site, as well as the site’s surrounding buffer zones. The anthropogenic light pollution research in recent decades reveals the problem is increasing around the world [78] confirming that only 22% of the environment in Europe remains free of light pollution [79]. Based on Table 5, it is evident that most of the time in summer on the chosen dates when the events in the evening and at night took place, the sky was cloudy. As ground-based illuminance measurements could not be performed on-site due to limited access and COVID-19 pandemic restrictions, we performed a simulation of artificial skyglow SRS data. This was achieved by taking into account the night sky brightness for the Wisłoujście Fortress (Table 1, point no. 1) from the LPM World Atlas, which is 18.52 mag/arc sec^2^, and converting it to an approximate total brightness of 4.22 mcd/m^2^ with the help of a calculator [80]. Using a simple transformation, we multiplied 4.22 mcd/m^2^ by 2π to obtain the illuminance (a perfectly diffused surface would reflect evenly over a 180-degree hemisphere, and there are 2π steradians in a hemisphere), which provided the value of 0.026 lx. Other research indicates that when there is extreme cloud cover, the average zenith luminance can reach a factor of 25 times the zenith luminance of a clear sky [81]. Assuming this value for our research, we estimated that the illuminance value during a cloudy night could reach 0.65 lx. This calculation is a significant simplification, but it allows the conclusion to be drawn that the Natura 2000 site should be monitored and that field measurements should be performed. This is because EUROBATS Guidelines no. 8 recommends no artificial lighting in the core of ecologically sensitive sites with the presence of bats and that any lighting for the buffer zone that surrounds Natura 2000 sites, including key feeding areas such as bodies of water, remain lower than 0.1 lx, with strict avoidance of direct ALAN [53]. These illuminance levels can be exceeded in this location on a regular basis on normal days without any nighttime event such as the Wisłoujście Festival.

The ground illuminance at the site on cloudy nights exceeds the EUROBATS recommendation on the basis of skyglow alone. Therefore, as city illumination breaches the conservation status of the Natura 2000 site, the situation argues for more aggressive policy controls in Gdańsk to reduce skyglow for the benefit of protecting the bat population.

In recent years, a new tool in the form of remote sensing has been applied to various research projects. For example, it was used to calculate an empirical environmental sustainability index [82], study global economic and demographic differences between countries [83], define global trends in exposure to light pollution [84], evaluate light pollution in global protected areas [85], model light pollution in suburban areas [86], estimate the effects of the COVID-19 lockdown on urban light emissions [87], assess the impact of ALAN on cancer risk in epidemiological studies [88], research methods for the assessment and monitoring of light pollution around ecologically sensitive sites [89], and additionally, facilitate the management of nesting sea turtles on the Mediterranean coastline affected by nighttime lighting [90]. There are also studies that have looked at bat activity for urban planning in three cities in France that are supportive of biodiversity. This involved remote sensing pictures taken by astronauts in the International Space Station. (These images are of much higher quality compared with the authors’ data.) This study also used ground-based data for the location of street lighting columns [91]. Another research project estimated the light pollution in urban and suburban regions of Pakistan with the help of satellite remote sensing (SRS) and geographic information system (GIS) techniques [92]. However, this study used data from a different satellite (DMSP), and the area was much larger, and therefore, the lower-resolution data meant the conclusions were sufficient. For epidemiological studies, some researchers believe that DMSP data should no longer be used [93]. Only one study could be identified that related to the ecological impact of ALAN that discussed general aspects of effective strategies and measures to deal with protected species and habitats [94].

While satellite remote sensing data create promising prospects to evaluate the impact of ALAN in natural environments and on endangered species, there was only one review study available on the opportunities of assessment and monitoring of ALAN around ecologically sensitive sites such as Natura 2000, with the help of remote sensing [95]. We postulate that this might be due to the fact that the obtained resolution was too low to analyse smaller areas to provide any meaningful conclusions.

Therefore, in this study, the authors concluded that the obtained remote sensing data (night lights estimated with satellite-based imagery) have their limitations, and thus they are an inappropriate tool for research on the Natura 2000 site, located in the Wisłoujście Fortress. Until there is a better data collection source in orbit, these studies are at a distinct disadvantage that can only be remedied by making in situ measurements on the ground.

As assessing the scale of light pollution is difficult to quantify, this issue is often disregarded by the conservation management of Natura 2000 sites. Together with other researchers [96], we argue that a new satellite mission optimised for observing night lights is urgently required to overcome these defects in available remote sensing imaging data.

The COVID-19 pandemic [97] and its effects are undoubtedly a huge challenge for modern-day communities all around the world, not only due to the many deaths that have occurred but also because of its impact on the natural environment. Due to lockdowns, we have witnessed for the first time just how the natural environment thrives during the day and night when human presence is curtailed and the associated anthropological burdens created in the last century are reduced [98]. There are studies that have identified the positive impact on air quality and climate from the reduction in human mobility on land and at sea due to enforcement [99], improved water quality [100], lower greenhouse gas emissions [101], less noise pollution [102], and the increased visibility of stars [103]. There are also positive reports connected to fauna. This includes wild bees as lockdowns have meant a number of insect-harming practices have been placed on hold [104], an increased number of turtle hatchlings in many countries where beaches were unoccupied [105], and instances where other animals have entered human settlements for the first time in years [106,107]. Unfortunately, there are not enough studies that have investigated the reduction of anthropogenic light pollution (ALP) to confirm how this has favourably affected ecosystems. While there are some studies that found a decrease [108,109,110], there are also studies that have identified an overall increase in ALP [111].

It would appear the lockdown enforced to help prevent the spread of COVID-19 in 2020 also had positive consequences for the endangered pond bat in the northern part of Poland, which resulted in an increase in their population. From the obtained information based on the annual work of bat researchers during the autumn bat swarming count on the Wisłoujście Fortress site, performed in 2020 during the night on 18/19 of September between the hours of 8 pm and 3 am, 93 bats in total were caught from five different bat species [64]. The most common among the caught species was *Myotis daubentonii*, which constituted 48% of all specimens, with *Natterera Myotis nattereri* accounting for 38% and *Myotis dasycneme* (pond bat) 10%. Apart from the already mentioned, there was also one specimen of a tiny *Pipistrellus pipistrellus* and one *Pipistrellus sp*. Fourteen pond bats were recorded and compared to previous years; this showed a 1.4-fold increase in their numbers. We believe that, due to the lockdown, nature in general, including bats, might have recovered from the constant presence of humans disturbing the bats’ summer colonies at Wdzydze Lake and that more bats were born in 2020. Therefore, more bats visited the site in September of 2020. This could also imply that if the Wisłoujście Festival had not taken place in August, bat researchers may have recorded even more bats during that time. Based on this observation, the authors believe that pond bats already visited this site in August for autumn swarming (Figure A1). This is also consistent with other existing research about pond bats in Europe, which confirms that, for the autumn migration period, they typically, around the middle of August, depart from their summer roosts to reach their hibernation sites in the second half of August.

Therefore, we believe there should be an investigation in order to establish if any events performed during the month of August that use artificial lights should be permitted on-site. The reason that only one pond bat was recorded in October 2020, during the second autumn bat count, would imply that due to the adverse impact of artificial lighting during the festival, the bats that were recorded in September never returned to the site, except for one. As pond bats are organisms that exhibit an adverse reaction to artificial lighting, ALAN cannot be ruled out as a contributing factor for their decline in the Natura 2000 area in recent years. Table 3 shows a positive possible correlation between the decline in the number of pond bats since 2016, since the first large-scale event with artificial lighting took place on-site. As no ground measurements were taken to assess the light exposures at the site before, during, and after the EDM festival, our data cannot establish it as the proximate cause, separate from the influence of noise or other factors associated with the event.

From the light pollution point of view, the existing regulation of the protection management plan for the Natura 2000 site Fortress Wisłoujście PLH220030 is a soft protection of Natura 2000, which explains why it achieves only marginal effectiveness. The reason for this is the legal nature of this act, which is a source of local law. This means the content and scope of the management plan are determined in detail by acts of higher legislative rank [112,113]. No provision in the Nature Conservation Act provides for specific protective measures against light pollution in the Natura 2000 protection management plan. However, this is part of a wider problem relating to the ineffectiveness of Polish law to counteract light pollution [114]. The Environmental Protection Law does not include excessive and harmful emissions of artificial light in terms of pollution at all. Consequently, the lack of systemic legal solutions certainly makes it difficult to protect Natura 2000 sites from the negative effects of artificial light at night. Rather, it is necessary to regulate it at the national level and to then provide environmental protection authorities with the necessary tools (tasks and competencies) to prevent, reduce, and control light pollution, especially in special sites such as Natura 2000. Based on the above example, it is clear that light pollution laws play a crucial role in overcoming this crisis in the near future. 

Counteracting artificial light pollution is not possible without proper legal regulations. Taking into account the importance of the problem and its universality, it seems that the effectiveness of the fight against light pollution should constitute an important element of the environmental protection policy at the European Union level. The analysis of Polish law from the perspective of the protection of Natura 2000 sites from light pollution leads to the conclusion that the system is currently inadequate. 

On the basis of the conducted interdisciplinary research, one can assume there are two conflicting interests on the site of the Natura 2000 Wisłoujście Fortress in Gdansk, Poland: nature-based versus human/cultural-heritage-based. The Regional Directorate for Environmental Protection in Gdańsk, as a non-unitary government administration body in the area of the voivodeship, is responsible for all matters related to the protection of the natural environment. This includes Natura 2000 sites such as, e.g., commissioning reports of the monitoring of wintering bats (with particular emphasis on the pond bat). The other party is the Museum of Gdańsk which acts as the administrator of the Wisłoujście Fortress complex and is responsible for the preservation of the historic monument itself. 

There is an obvious need to increase awareness of the active protection of bats by the Museum of Gdańsk. Considering what researchers know about the adverse effects of light pollution on bats, it is unreasonable that a large-scale music event such as the Wisłoujście Festival has been allowed each year since 2018 (sometimes for up to three continuous nights until the early morning hours) in the Natura 2000 area when the autumn bat swarming begins in late August (see Appendix A, Figure A1) without proper research being undertaken. 

We believe the problem stems in part from a lack of funds. (The Museum as an administrator must use every opportunity to generate income in order to perform the next steps of the reconstruction and refurbishment of the architectural complex.) Further complicating the situation is a possible lack of clear communication, along with a lack of information knowledge transfer between the two bodies about the Natura 2000 protection management plan. This includes the scope of permitted activities on-site and the consequences of undertaking activities that may have a significant negative impact on the conservation objectives of Natura 2000 sites (see Appendix D).

## 7. Limitations of the Study

The final results of the study mentioned in Section 5 are influenced by the study’s limitations, which are related to the characteristics of satellite imagery and remote sensing analysis, identified in the following three areas.

### 7.1. Research Methodology Issues

The indicated limitations translate directly into the limitations related to the use of the web services https://www.lightpollutionmap.info/ and https://lighttrends.lightpollutionmap.info/ (accessed on 5 May 2021) because the source data for these portals are data from the VIIRS sensor (see Section 5.3 and Appendix C). In addition, it should be noted that the compositions shared by both web portals are the result of processing rasters by a complex algorithm that differ from each other. This means the output data are also different for the same measurement periods. 

### 7.2. Data Issues

• The main constraints are the lack of registrations on each day of the year. For instance, in the analysed area, there is a gap between week 16 and week 34 and low-quality registrations.
• Remote sensing data provide a global radiance value (direct and reflected light emissions) perceived from space for a pixel, which is imprecise compared to ground-based measurements [91].

### 7.3. Infrastructure Issues—Collection Method

For the type of sensor, which was used in these analyses, the following factors were important: 

• The limited spectral range of the radiometer sensor.

The satellite sensor captures nocturnal images in the limited spectral response range of 505–890 nm. This range does not match the visible spectrum of 380–750 nm, which is detected by the human eye. In addition, the satellite sensor does not register light emitted by blue-rich white light sources such as metal halides and white-light-emitting diodes (LEDs), both of which have a higher content of blue and green wavelengths of light. This does not allow for a full analysis of visible light (violet, part blue). However, the satellite sensor can measure infrared wavelengths such as bush fires [115];

• Cloud cover.

In addition, most of this radiation is unable to pass through clouds (it is reflected, absorbed, and scattered) and therefore cannot be accurately measured.

• Error measurements of the areas located in coastal cities.

Due to the man-made skyglow phenomenon (which the CIE defines as attributable to anthropogenic sources of radiation, e.g., outdoor lighting, which includes radiation that is emitted directly upwards, as well as radiation that is reflected from the surface of the Earth [116]), there is an error in the measurement of unilluminated areas which are located near brightly lit coastal cities such as Gdańsk [117,118], as satellites still record light emissions from these areas.

• Registration angle.

Often this angle deviates from the nadir registration, which would provide the best results for the acquisition of upwards-emitted radiation. Changing the registration angle only records the light sources emitted and scattered towards the sensor, but the change of such an angle also causes the light sources to be obscured by other objects that have a higher position.

• Registration of other phenomena that are an indirect result of the emission of natural light on Earth, which is visible from space.

This phenomenon includes the emission of light from the stars, sunlight reflected from the moon, and the emission from the aurora borealis, airglow, or fires. In addition, it should be taken into account that the mentioned sources of electromagnetic wavelengths and the emitted artificial light from the Earth are subject to certain phenomena in the atmosphere (absorption, scattering, reflection), and this causes distortion, which provides unclean data on only the direct emission of artificial light from sources on the ground.

• Registration time.

The registration made by the VIIRS system takes place around 1:30 a.m./2:30 a.m. (summer time). This time of night does not allow for the registration of light emitted by outdoor dining premises and entertainment centres because often these places do not operate after midnight, which means they also usually turn off unnecessary outdoor lighting.

• Seasonality of measurements.

Measurements are limited when areas are covered with snow. However, at high latitudes (such as the analysed area), measurements are not made during the summer period (which could be relevant for the analysis carried out in this paper) due to the likelihood of highly scattered light causing inaccurate measurements by the sensor.

## 8. Conclusions

In this paper, we used the Natura 2000 site at the Wisłoujście Fortress in Gdansk, Poland, as a case study to investigate the current protection of wildlife from the harm of exposure to artificial light at night (ALAN), in particular, the disruption to pond bats (*Myotis dasycneme*) from nighttime events including an annual music festival held at this location each August. We presented satellite remote sensing data to identify changes in the upwards radiance during the past decade, as well as counts of bats seen at the site during the same time period. An observed decline in bat counts was confirmed during a period of time in which remotely sensed upwards radiance in and near the site partially increased. Along with an analysis of the available legal mechanisms for the protection of the site and noted shortcomings, we concluded that the reported bat decline may be related to the music festival. While there was an increase in bats seen in autumn 2020, this most likely stemmed from changes in human social behaviours and activity during lockdowns during the COVID-19 pandemic. We argue for a change in conservation practices at the site following these observations as well as a need to modify existing laws and regulations to provide better protection for this and similar locations.

In 2013, the European Union adopted the Green Infrastructure policy to support ecological corridors and landscape connectivity beyond Protected Areas such as Natura 2000 sites and to maintain healthy ecosystems by creating a network of natural and semi-natural areas in urban and rural environments. This should help support the preservation and sustainable use of land as well as the management of land [119,120].

**Figure 20:**
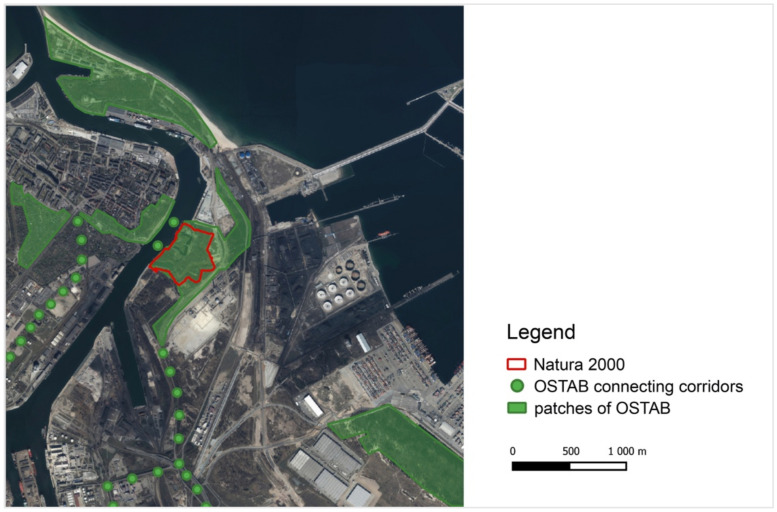
The Natura 2000 Wisłoujście Fortress with identified OSTAB elements including the OSTAB core nature matrix and OSTAB connecting corridors. Source: authors’ elaboration based on [121].

Based on these requirements, the city of Gdańsk established a city-wide urban system of biologically active areas called “OSTAB” [121], and the Natura 2000 Wisłoujście Fortress is an important part of this network (Figure 20), even though its role seems to have been evidently overlooked. OSTAB consists of three elements—OSTAB core nature matrix, patches of OSTAB, and OSTAB connecting corridors. The OSTAB concept should be evaluated positively, but until it is reflected in concrete planning solutions, it cannot be considered as a desirable form of nature conservation and ecological balance.

Research has indicated that reducing light pollution levels in dark corridors improves the connectivity for bats in urban landscapes [122,123]; furthermore, the existence of dark corridors such as rivers and patches of vegetation in human-inhabited landscapes appears to be crucial in successfully restricting the adverse effects of ALAN on biodiversity involving insects that bats feed on [124]. We believe that adhering to this approach will make the core area of Natura 2000 protection sites more resilient and supportive to biodiversity.

Ensuring the effectiveness of pond bat conservation depends on the introduction of appropriate regulations. Firstly, it is necessary to indicate the environmental requirements in national law. Secondly, the authorities responsible for carrying out conservation tasks in Natura 2000 sites must be provided with the means to control and monitor the level of light pollution, as well as be able to take necessary action against the violation of lighting standards. Most Natura 2000 places experience light pollution impacts but this type of pollution is not even listed on their standard forms.

The same applies to the protection management plan for the Natura 2000 Wisłoujście Fortress site PLH220030. When counteracting light pollution, the objectives of the protection tasks specified in this act cannot be effectively realised without legal tools enabling the rational planning of outdoor lighting. Only appropriate planning tools, either in the form of a separate urban lighting masterplan [125] or using a legal resolution on the landscape (pl. uchwała krajobrazowa [126]) adopted by the local authorities, can be effective in reducing uncontrolled ALAN emissions in sensitive species protection areas, such as Natura 2000 sites.

In addition, recent studies indicate that ALAN can negatively impact various nocturnal organisms including bats and this possible anthropogenic factor has often been overlooked [127,128], especially with the widespread use of LEDs for outdoor lighting which might lead to a serious decline in the pond bat. Therefore, an investigation via a systemic, interdisciplinary approach [129,130] seems to be the only viable solution for such a complex undertaking to investigate all of the different aspects involved in species decline, including ALAN.

Further studies are necessary for conservation management and to determine the actions needed to be performed. This should involve five experts including (1) a lighting specialist, (2) a low-level aerial remote sensing specialist, (3) a bat specialist, (4) an environmental acoustic engineer, and (5) a lawyer. In addition, it is imperative to ensure the development, testing, and dissemination of best lighting practices in the design and implementation of biodiversity policies and legislation, as well as facilitating the continual improvement of knowledge in this field take place.

As the next electronic music Wisłoujście Festival is due to happen in August 2022, we recommend environmental lighting impact assessments (ELIA) be made before, during, and after the festival to monitor the effects of human disturbance (light and sound) on pond bats.

Additionally, this research would benefit significantly from in situ ground-based light pollution measurements during the festival to provide more context to the remotely sensed radiances on which our study depends so strongly. This could include applying other tools such as a night sky brightness meter Sky Quality Meter (SQM) or Sky Sensor (TESS-W), illuminance meter, and an RGB digital single lens reflex (DSLR) camera to record and investigate the illumination on-site.

This will also provide insights to help improve the planning of similar events in sensitive sites in Europe to ensure such vulnerable species are adequately protected from ALAN. The enhancement of living conditions for bats will assist in boosting their declining numbers and, consequently, increase the local population of these endangered mammals.

Museum of Gdańsk recently announced that, after the 2022 reconstruction works are completed, the Wisłoujście Fortress will be opened to the public all year long. This means it is of significant importance to update the protection management plan for the Natura 2000 site Fortress Wisłoujście PLH220030 based on the in situ measurements before and after the EMD festival and the results of the ELIA.

In 2020, due to the lack of proper lighting regulatory frameworks to reduce light pollution, the International Commission on Illumination (CIE) founded a new Technical Committee TC 4-61 called “Artificial lighting and its impact on the natural environment” [131]. Its purpose is to provide guidance to minimise the effects of artificial lighting on the natural environment, including impacts on flora and fauna. It is hoped that this research work will encourage improved recommendations by this technical committee to be established in the near future for the specific habitats of endangered bats and other organisms, including Natura 2000 sites located adjacent to urban environments.

## Figures and Tables

**Table 1:** Geographical coordinates of the seven analysed points. Source: authors’ elaboration.

Point Number	Geographical Coordinates	
1	54.39530, 18.68005	Wisłoujście Fortress (pl. Twierdza Wisłoujście)
2	54.38898, 18.66985	Przeróbka district, near the mining pool (pl. Dzielnica Przeróbka okolice basenu górniczego)
3	54.39209, 18.65740	Outskirts of the Letnica district (pl. Obrzeża dzielnicy Letnica)
4	54.40069, 18.67095	The Nowy Port district (pl. Dzielnica Nowy Port)
5	54.40234, 18.68596	Outskirts of the Gdańsk Shipyard (pl. Obrzeża Portu Północnego)
6	54.40530, 18.71971	The Northern Port area (pl. Port Północny)
7	54.38540, 18.71886	The DCT Gdańsk S.A. container terminal (pl. DCT terminal Kontenerowy)

**Table 2:** Type of land cover within the Natura 2000 Wisłoujście Fortress based on BDOT10k. Source: authors’ elaboration.

Level 2		Area (m^2^)	Level 3		Area (m^2^)
Code	The Name of the Object		Code	The Name of the Object	
PTWP	surface water	43,811.78	PTWP02	flowing water	43,811.78
PTZB	building	15,978.81	PTZB03	industrial and storage buildings	4121.74
			PTZB05	remaining buildings	11,857.07
PTLZ	forest and wooded area	75,875.17	PTLZ01	forest	75,875.17
PTTR	grassy vegetation and agricultural cultivation	26,064.04	PTTR01	grassy vegetation	26,064.04
Sum					161,729.80

**Table 3:** An overview of the number of individual pond bats (*Myotis dasycneme*) between 2011 and 2020 during the autumn swarming season count on-site in October [64]. This suggests there is a correlation between the daytime and nighttime light, sound, and music events organised on-site, or on the adjacent river, and a reduction in pond bat numbers. Source: authors’ elaboration.

Year	Number of Pond Bats	Daytime Event ^1^	Nighttime Event ^2^
2011	5	+	
2012	7	+	
2013	4	+	
2014	9	+	
2015	8	+	
2016	0		+
2017	0		+
2018	0		+
2019	0		+
2020	1		+

^1,2^ For the full list of daytime and nighttime events, please see Appendix A, Table A1.

**Table 4:** Comparison of data from two services—absolute values of differences (unit nW/cm^2^·sr). Source: authors’ elaboration.

Point		Year							Value Differences in Point Measurements		
		2014	2015	2016	2017	2018	2019	2020	Average	Minimum	Maximum
1		8.95	2.93	0.96	2.01	11.72	1.51	2.58	4.38	0.96	11.72
2		12.93	0.52	0.46	4.52	2.67	1.21	0.49	3.26	0.46	12.93
3		16.61	18.33	2.16	2.52	7.90	2.17	0.25	7.13	0.25	18.33
4		11.06	5.94	3.50	1.94	4.40	3.90	0.75	4.50	0.75	11.06
5		2.33	0.53	3.71	1.03	1.52	0.49	1.48	1.58	0.49	3.71
6		2.64	5.40	3.10	0.28	1.30	2.24	4.32	2.75	0.28	5.40
7		5.63	5.10	0.88	2.49	8.06	1.91	17.32	5.91	0.88	17.32
Value differences in annual analyses	Average	8.59	5.54	2.11	2.11	5.37	1.92	3.88	4.22	-	-
	Minimum	2.33	0.52	0.46	0.28	1.30	0.49	0.25	-	0.25	-
	Maximum	16.61	18.33	3.71	4.52	11.72	3.90	17.32	-	-	18.33

**Table 5:** List of dates of events including nighttime illumination organised in the area of the Wisłoujscie Fortress together with the availability of the analysed data. Source: authors’ elaboration.

The Date of the Event dd/mm/yyyy	Nighttime Events	Data			
		Date	Description	Characteristic	Possibility of Use
13/08/2016	Naval Battle. Secrets of the Wisłoujście Fortress	11/08/2016	VNP46A2.A2016224	No data coverage	-
		12/08/2016	VNP46A2.A2016225	No data coverage	-
		13/08/2016	VNP46A2.A2016226	Very low quality	No
25–26/08/2018	Festival Wisłoujście	24/08/2018	VNP46A2.A2018236	Highly cloudy	No
		25/08/2018	VNP46A2.A2018237	Highly cloudy	No
		26/08/2018	VNP46A2.A2018238	No data coverage	-
		27/08/2018	VNP46A2.A2018239	High quality	Yes
09–11/08/2019	Festival Wisłoujście	08/08/2019	VNP46A2.A2019220	No data coverage	No
		09/08/2019	VNP46A2.A2019221	No data coverage	No
		10/08/2019	VNP46A2.A2019222	No data coverage	-
		11/08/2019	VNP46A2.A2019223	No data coverage	-
		12/08/2019	VNP46A2.A2019224	Very low quality	No
21–23/08/2020	Festival Wisłoujście	21/08/2020	VNP46A2.A2020234	No data coverage	No
		22/08/2020	VNP46A2.A2020235	Very low quality	No
		23/08/2020	VNP46A2.A2020236	Very low quality	No
28–30/08/2020	Festival Wisłoujście	27/08/2020	VNP46A2.A2020240	High quality	Yes
		28/08/2020	VNP46A2.A2020241	High quality	Yes *
		29/08/2020	VNP46A2.A2020242	Very low quality	No
		30/08/2020	VNP46A2.A2020243	Very low quality	No
		31/08/2020	VNP46A2.A2020244	Very low quality	No

* There was one night where it was possible to verify whether the lights from the festival were registered.

## Appendix A. An Overview on Bats in the Context of ALAN

### Appendix A.1. Bats—An Overview

In Europe, bats are strictly protected in order to prevent a decrease in their populations. This decline has been linked to a reduction in foraging habitats and/or a decrease in available roosting, combined with a lack of overwintering locations that are free from human disturbance. Bats are the only mammals that can fly, and these species are important natural predators for the world (nighttime insect hunters), as their presence makes it possible to limit the use of toxic insecticides in agriculture and forests [132]. They also eat mosquitos which spread various mosquito-borne diseases to humans and livestock.

When the living ecosystem of bats is strong, healthy, and stable, they thrive as a species. However, a reduction in their numbers is a natural marker of the state of the entire ecosystem and an indication of a serious problem that needs to be addressed [133]. As female bats produce only one to a maximum of two offspring per year, this makes them highly vulnerable to extinction.

Bats are divided into two groups based on their size and whether they use echolocation and other specific features: (1) megabats (Megachiroptera) and (2) microbats (Microchiroptera), with the latter being smaller in size and more diverse, who use echolocation for orientation and prey. Microbats also have smaller eyes, and their teeth may vary in size and shape, depending on the type of food they consume.

Many European bats hibernate, migrate, or exhibit a mixture of both behaviours throughout winter to survive harsh outdoor conditions. Some roost in trees, but often due to a lack of such habitats, they use man-made environments such as the attics of old barns, abandoned buildings, or church spires, as well as mines, caves, bunkers, and cellars of historic buildings and structures.

Bats that live at high and mid-latitudes in North Europe typically hibernate up to six months, as during the winter months, the insects they rely upon for food are scarce or non-existent. Hibernation allows bats to survive this period of time as their metabolic functions slow down in order to conserve energy. For instance, their heart rate drops, and body temperature is lowered.

Bats use echolocation to “see” in the dark by producing high-frequency sound waves through their mouth or nostrils to navigate and locate their food. When these waves bounce off objects in their environment, bats listen to the echo which allows them to orientate themselves and locate their prey. Until recently, researchers believed bats used short-distance flights during foraging, hunting, the change of roosts, and social behaviour. It was assumed that they do not migrate and that the lifestyle of bats is sedentary. However, it has been discovered that some bats migrate like birds do, based on the seasons [134]. For some, it involves a local migration of around 10 km to long-range distances up to hundreds of kilometres. A behaviour called natal philopatry [135], where animals come back to their birthplace to breed, can be observed in adult bats when they return to their summer and winter roosts year after year. At the end of spring, female bats gather in larger groups to roost, where they give birth and take care of their young newborn bats.

In the early autumn, bats gather to create a swarming flight movement forming a large or dense group. This behaviour is thought to primarily serve as a mating system, but it may also be associated with helping young bats to learn the location of their winter hibernacula site [31].

### Appendix A.2. Bats and ALAN

Most bats are nocturnal animals [136], and therefore, they forage in the darkness away from illuminated conditions. They have rods in their eyes which are sensitive to minimal illumination [137], and bats can detect basic visual cues and motion. Like all nocturnal living organisms, these mammals evolved with exposure to very low levels of natural light from the moon, stars, and planets and twilight conditions. For instance, even the light reflected from the full moon which has a low-level illuminance between 0.05 to 0.1 lx [138] can alter the physiology of bats. In addition, when there is an increase in light levels, some bats use their vision instead of echolocation [139]. There are also bats that have colour vision [140] and a few that can even perceive ultraviolet lighting [141].

Bats also react to artificial light (intensity, spectral power distribution, direction, and/or its duration) primarily by changing their behaviour. This can involve escaping [142], reducing activity [143], changing flight routes to feeding grounds [144], and avoiding hunting [145] or hunting insects that concentrate around artificial light sources [146]. There are some bats that forage in illuminated areas near street lighting, where they can easily locate the insects that they prey on. However, this visibility also makes the bats themselves vulnerable to other predators such as cats and owls [147], which can disrupt the food chain.

The introduction of light sources such as metal halide (MH) and light-emitting diodes (LEDs), which both emit high levels of disruptive short wavelengths of light in their spectrum, is found to attract more insects [148,149]. This type of lighting will also adversely suppress the melatonin levels of bats and, as a consequence, disrupt their biological clock [19]. This is in line with research that confirms light pollution is a biodiversity threat for many organisms, including bats [150].

**Table A1:** An overview of the daytime and nighttime light and sound events between 2011 and 2020, organised on the site of the Natura 2000 Wisłoujście Fortress or on the adjacent river. Source: authors’ elaboration.

Year	Daytime Event	Nighttime Event
2011	Naval Battle. Defence of the Wisłoujście Fortress (pl. Bitwa Morska. Obrona Twierdzy Wisłoujście) held on 16.04.2011. First edition	-
2012	Naval Battle. Defence of the Wisłoujście Fortress (pl. Bitwa Morska. Obrona Twierdzy Wisłoujście) held on 06.05.2012	-
2013	Naval Battle. Defence of the Wisłoujście Fortress (pl. Bitwa Morska. Obrona Twierdzy Wisłoujście) held on 06.07.2013	-
2014	Naval Battle. Defence of the Wisłoujście Fortress (pl. Bitwa Morska. Obrona Twierdzy Wisłoujście) held on 26.07.2014	-
	Naval Battle on the Motlau River during the Baltic Sail event. Event moved to a different location held on the river on 6.07. 2014	
2015	Naval Battle on the Motlau River during the Baltic Sail event. Event moved to a different location on the river, held on 4.07. 2015 at 20:15–21:00	-
2016	Naval Battle on the Motlau River during the Baltic Sail Gdańsk event. Event held in a different location on the river on 3.07.2016	Evening sound and light show: Naval Battle. Secrets of the Wisłoujście Fortress (pl. Bitwa Morska. Tajemnice Twierdzy Wisłoujście) held on 13.08.2016 at 9:30 p.m. [152,153]
2017	Closing event held on 30.09.2017 with the firing of artillery of cannons	Naval Battle on the Motlau River during the XXI Baltic Sail Gdańsk event. Event moved to a different location on the river on 1.07.2017 [154]
2018	Naval Battle. Defence of the Wisłoujście Fortress (pl. Bitwa Morska. Obrona Twierdzy Wisłoujście) held on 07.07.2018	Daytime and nighttime annual EDM Festiwal Wisłoujście, 1st edition held on 25–26.08.2018 [155]
	Closing event. (pl. Pożegnanie lata w Twierdzy Wisłoujście) held on 30.09.2018	
2019	Naval and Land Battle Wisłoujście 1577. Wisłoujście Fortress (pl. Bitwa Morska i Lądowa Wisłoujście 1577) held on 06.07.2019	Daytime and nighttime annual EDM Festiwal Wisłoujście, 2nd edition held on 9–10.08.2019 [156]
2020	The COVID-19 lockdown caused the cancellation of most events	Daytime and nighttime annual EDM Festiwal Wisłoujście, 3rd edition held on 21–23.08.2020 and 28–30.08.2020 [157]

## Appendix B. Regulatory Frameworks for ALAN and Bats

**Table A2:** Examples of regulatory frameworks on ALAN in the context of environmental protection and bats. Source: authors’ elaboration.

Category	Year	Name	Country of Origin
Guidelines	2001	Bats in Forests—Information and recommendations for forest managers by the Federal Agency for Nature Conservation (Bundesamt für Naturschutz—BfN) and the German Association for Landcare (Deutscher Verband für Landschaftspflege—DVL) [158]	Germany/EU
	2006	Bats In Houses—Guidance for Householders by the Irish National Parks and Wildlife Service (NPWS) in cooperation with Bat Conservation Ireland [159]	Ireland
	2006	Bat Roost in the Alpine Area: Guidelines for Renovation of Buildings by the Centre for Bat Conservation and Research in Austria (KFFÖ) [160]	Austria
	2009	Artificial Light in the Environment Report by the Royal Commission on Environmental Pollution [161]	UK
	2011	Building Bat Friendly: information for home owners, architects and policy officers by Landschapsbeheer Flevoland, the Dutch Mammal Society and TAUW [162]	NL/EU
	2012	Landscape and urban design for bats and biodiversity by Bat Conservation Trust [163]	UK
	2013	PLG04 Guidance on undertaking environmental lighting impact assessments by the Institution of Lighting Professionals (ILP) [164]	UK
	2014	Nature-friendlier lighting of objects of cultural heritage (churches) by Dark-Sky Slovenia [165]	Slovenia
	2016	CIBSE LG06/16. Lighting Guide 6: the exterior environment by the Chartered Institution of Building Services Engineers (CIBSE) and Society of Light and Lighting (SLL) [166]	UK
	2017	CIE 150:2017. Guide on the Limitation of the Effects of Obtrusive Light from Outdoor Installations, 2nd Edition by the International Commission on Illumination (CIE) [167]	International
	2017	EUROBATS guidelines no. 5. Guidelines for Surveillance and Monitoring of European Bats, 3rd edition by the United Nations Environment [168]	EU/International
	2017	EUROBATS guidelines no. 4. Protection of overground roosts for bats by the United Nations Environment [169]	EU/International
	2018	GN08/18. Guidance Note 8 for Bats and Artificial Lighting by the Institution of Lighting Professionals (ILP) [170]	UK
	2018	EUROBATS guidelines no. 8. Guidelines for consideration of bats in lighting projects by the United Nations Environment [53]	EU/International
	2018	WaterSpace Design Guidance. Protecting Bats in Waterside Development by the Planning Department of Bath and North East Somerset Council [171]	UK
	2019	Designing for Biodiversity. A Technical Guide for New and Existing Buildings by Bat Conservation Trust and RIBA Publishing [172]	UK
	2019	EUROBATS guidelines no. 9. Guidance on the conservation and management of critical feeding areas and commuting routes for bats by the United Nations Environment Programme [173]	EU/International
	2020	GN01/20. Guidance Note 1 for the reduction of obtrusive light 2020 by the Institution of Lighting Professionals (ILP) [174]	UK
Procedures	2000	Action Plan for the Conservation of the Pond Bat in Europe: *Myotis Dasycneme* (Nature and Environment) by the Council of Europe [175]	International
	2004	Bat Workers’ Manual by the Joint Nature Conservation Committee JNCC [176]	UK
	2010	EUROBATS guidelines no. 2. Protecting and managing underground sites for bats by the United Nations Environment Programme [177]	EU/International
	2011	Model Lighting Ordinance (MLO)—User’s Guide by the International Dark-Sky Association (IDA) and Illuminating Engineering Society of North America (IESNA) [178]	USA/International
	2015/2019	A guide to the implementation of the Agreement on the Conservation of Populations of European Bats (EUROBATS) [179]	EU
	2018	Action Plan for the Conservation of All Bat Species in the European Union 2018–2024 by European Commission [180]	EU
Standards and Codes	2012	The SLL Code for Lighting by The Society of Light and Lighting (SLL) [181]	UK
	2003/2015	EN 13201-2: Road lighting—Part 2: Performance requirements by the European Committee for Standardisation (CEN) [182]	EU
	2014	EN 12464-2 Light and Lighting—Lighting of Work Places, Part 2: Outdoor Work Places by the European Committee for Standardisation (CEN) [183]	EU
	2020	ANSI/IES LP-11-20 Environmental Considerations for Outdoor Lighting by the American National Standards Institute and Illuminating Engineering Society [184]	USA/International

## Appendix C. A Short Overview of the DMSP-OLS and Suomi NPP-VIIRS Satellites/Sensors

### Appendix C.1. The Operational Linescan System Sensor

The Operational Linescan System sensor on board the Defence Meteorological Satellite Program weather satellites (in short DMSP-OLS) [185] was first established in 1962 as a classified military mission of the satellites Defence Satellite Application Program (DSAP). Ten years later, information on its existence was given to the general public, including remote sensing researchers. Its main aim was/is to observe meteorological (clouds), oceanographic, and solar-terrestrial physics for the United States Department of Defence. The program is managed by the United States Space Force with on-orbit operations provided by the National Oceanic and Atmospheric Administration (NOAA). This satellite is equipped with an optical-mechanical scanner that can collect data in a few different spectral bands of the electromagnetic spectrum. It provides daily global measurements of visible light and near-infrared (NIR) light when the satellite passes approximately at 8.30 a.m. The spatial resolution of the DMSP-OLS data is constant throughout the entire imaging belt and amounts to 2.70 km per pixel globally and 0.55 km per pixel locally [186,187]. There are two dataset types available from DMSP-OLS: (1) time-series dataset and (2) radiance-calibrated datasets. The first World Atlas of artificial night sky brightness [188] was based on information provided by DMSP-OLS.

### Appendix C.2. The Visible Infrared Imaging Radiometer Suite Sensor

The Visible Infrared Imaging Radiometer Suite sensor on board the weather satellite Suomi National Polar-orbiting Partnership (in short Suomi NPP-VIIRS) [189] is a joint mission of the National Oceanic and Atmospheric Administration (NOAA) and National Aeronautics and Space Administration (NASA).

This was one of the first satellites launched in October 2011 to provide improved parameters in sensor resolution and calibration to conduct quantitative light pollution studies, as it has a ground footprint of 3060 km at the satellite’s average altitude of 824 km [190]. This satellite is equipped with an optical-mechanical scanner that can collect data in 22 different spectral bands of the electromagnetic spectrum. One of them, DNB (Day–Night Band), provides daily global measurements of visible light and near-infrared (NIR) light when the satellite passes approximately at 1.30 a.m. The high sensitivity of the DNB in lowlight conditions allows for nighttime analysis. The spatial resolution of the VIIRS-DNB data is constant throughout the entire imaging belt and amounts to 0.74 km per pixel. One of the greatest advantages of using this technology is the nature of the information itself about the intensity of light at night. Due to the appropriate calibration, the measurement data are reliable. As the instrument is a radiometer, the intensity of light is measured in radiometric values (nW/cm^2^·sr).

**Table A3:** The key differences between the DMSP-OLS and Suomi NPP-VIIRS satellites/sensors which both provide free-of-charge data based on [57,191,192]. Source: authors’ elaboration.

Category	DMSP	Suomi
Sensor	OLS	VIIRS-DNB
Accessibility	Free of charge	Free of charge
Availability of data in years	1992–2013	2012.04–2021.03
Data coverage	Annually	Annually, monthly, daily
Spectral bands	400–1100 nm	505–890 nm
Image resolution	30 arcsecond/~1 km at the Equator	15 arcsecond/~500 m at the Equator (VNP46)
Radiometric resolution	6 bit	14 bit
Swath width	2964 km	3040 km
Available at	https://www.ngdc.noaa.gov/eog/download.html (accessed on 5 May 2021)	https://www.ngdc.noaa.gov/eog/download.html (accessed on 5 May 2021) https://ladsweb.modaps.eosdis.nasa.gov/archive/allData/5000/VNP46A2/ (accessed on 5 May 2021)

### Appendix C.3. Interactive Web Mapping Services to Visually Show Light Pollution Levels

• Light Pollution Map (LPM) [193]

This interactive website permits users to generate graphs (a colour-coded projection onto a map of the Earth) which demonstrates over time the annual changes in the amount of upwards-directed light for any given location worldwide. The LPM provides information on the increasing radiance—the brightness of the sky in real units. Winter months are usually excluded from the analysis if there is snow in certain areas. This service uses information from VIIRS (2012–2020), the World Atlas (2015), and the location of IAU professional and amateur astronomical observatories, as well as recording clouds (from EUMETSAT, NOAA, MOSDAC/SAC/ISRO, and CIRA), the Aurora 1 h forecast (from NOAA), and Sky Quality Meter (SQM) and Sky Quality Camera (SQC) measurements. It also allows for distance and azimuth measurements.

• Radiance Light Trends (RLT) [194]

RLT is similar in concept to the LPM, but it is designed for more advanced users, as this application allows the verification of average monthly data, not just annual data for a selected region or a site, in order to analyse changes in light emissions. It uses information from two US-based satellites/sensors: the DMSP-OLM and Suomi NPP-VIIRS.

Both services use Microsoft’s interactive Bing Map as an overlay platform, and they vary in three major aspects listed in Table A4.

**Table A4:** The key differences between the Light Pollution Map and Radiance Light Trends (RLT) web mapping services. Source: authors’ elaboration.

Category	Light Pollution Map (LPM)	Radiance Light Trends (RLT)
VIIRS overlays *	Average combined	Median combined
Outlier **	No outlier removal	Outlier removal
Calibration	Zero-point calibration	With or without zero-point calibration

* *VIIRS* image *overlaid* on interactive maps. ** a data point that differs significantly from other observations.

• NASA’s Black Marble [195]

To monitor and evaluate the detailed daily, seasonal, and annual variations of light pollution for scientific observations that require much higher resolution images and data, NASA has developed a Black Marble product suite based on VIIRS-DNB, with data that are calibrated, corrected, and validated on a daily basis [196].

The current available VNP46A2 dataset (VIIRS/NPP Gap-Filled Lunar BRDF-Adjusted Nighttime Lights Daily L3 Global 500 m Linear Lat Lon Grid) stands for the corrected daily moonlight and atmosphere nighttime light (NTL) data, available at 500 m spatial resolution. This specific product has seven layers that contain information on BRDF-corrected NTL, which are daily compositions that take into account the influence of the atmosphere and the lunar phase of the moon in order to remove the influence of extraneous artefacts and biases [197].

## Appendix D. The Natura 2000 Wisłoujście Fortress Site PLH220030 and Its Protection Management Plan

The Natura 2000 Wisłoujście Fortress site PLH220030 was established in 2017 on the basis of the ordinance of the Minister of Environment of the Republic of Poland. The area is recognised as a special area of protection of habitats to permanently protect populations of species other than birds, endangered with extinction in order to restore the proper conservation status of these species [198].

The most important legal act regulating the principles and activities of nature protection in the analysed area is the Order of the Regional Director for Environmental Protection in Gdańsk, with the establishment of a protection management plan for the Natura 2000 site Fortress Wisłoujście PLH220030 [199]. Annex 3 identifies existing and potential threats to the favourable conservation status of animal species and their habitats. The object of protection is the pond bat species (*Myotis dasycneme*), which is subject to specific legal protection provided for by the following:

1. The Habitats Directive: Annex I (as an animal species of Community interest whose conservation requires the designation of special SAC) and Annex IV (as an animal species of Community interest in need of strict protection);
2. The Bern Convention on the Conservation of European Wildlife and Natural Habitats: Annex II (Strictly protected fauna species);
3. The Convention on the Conservation of Migratory Species of Wild Animals: Annex II (as migratory species requiring international cooperation);
4. The Agreement on the Conservation of Populations of European Bats (EUROBATS) (as an animal species occurring in Europe);
5. Ordinance of the Minister of Environment on the protection of animal species: Annex IV (as an animal species requiring the establishment of zones for the protection of sanctuaries, breeding sites, or regular places of residence and their size);
6. The pond bat species (*Myotis dasycneme*) is also on the International Union for Conservation of Nature (IUCN) Red List of Threatened Species, classified as “near-threatened” (NT) [46]. This means the species is close to being at high risk of extinction in the near future.

Threats to the pond bat in the analysed Natura 2000 site are land use and siting, renovation and the development of bastions, the removal of micro-shelter including the removal of old trees, the proximity of “Siarkopol” factory and the contamination of soil and water with sulphur, and roads and highways (Sucharski Route) potentially on the bats’ commuting routes between summer and winter hiding places in spring and summer. This location exposes the bats to possible artificial light pollution. Therefore, Annex 5 of the Natura 2000 protection management plan [200] identifies activities related to the maintenance or modification of the management practices for the protected site. Firstly, there is an annual lighting restriction during the months of September through to November (switching off the lights during the bat swarming period); however, these restrictions are not adhered to beyond this time in spring and summer. In addition, there are site-specific restrictions during this special period, such as switching off the lights from dusk to dawn in the South-Eastern Bastion and the Ostroróg Bastion (obligatory measures) and reducing artificial lighting at night inside the shelters in the Artillery Bastion and the Water Gate Bastion. Night lighting allowance inside the casemates is limited to once a week.

During the bats’ hibernation period (October to March), the lights should also be switched off to avoid disturbing this protected species. Additionally, to reduce disturbance to bats during hibernation, the entry of tourists and renovation works are restricted. The entity responsible for these conservation tasks is the Museum of Gdańsk [201], previously known as Gdańsk City Museum, as the Fortress is its branch.
